# An annotated type catalogue of seven genera of operculate land snails (Caenogastropoda, Cyclophoridae) in the Natural History Museum, London

**DOI:** 10.3897/zookeys.842.29243

**Published:** 2019-05-07

**Authors:** Chirasak Sutcharit, Jonathan D. Ablett, Somsak Panha

**Affiliations:** 1 Animal Systematics Research Unit, Department of Biology, Faculty of Science, Chulalongkorn University, Bangkok 10330, Thailand Chulalongkorn University Bangkok Thailand; 2 Division of Higher Invertebrates, Natural History Museums, London, SW7 5BD, UK Natural History Museums London United Kingdom

**Keywords:** systematics, type specimen, Southeast Asia, taxonomy, NHM, molluscs, conservation

## Abstract

The collection of the seven cyclophorid snail genera housed in the Natural History Museum, London (NHM), includes 95 available species-level names belonging to the genera *Pterocyclos* Benson, 1832, *Cyclotus* Swainson, 1840, *Myxostoma* Troschel, 1847, *Rhiostoma* Benson, 1860, *Scabrina* Blanford, 1863, *Crossopoma* Martens, 1891, and *Pearsonia* Kobelt, 1902. Lectotypes are here designated for twelve available species-level names to stabilise existing the nomenclature. A complete catalogue of these types, including colour photographs, is provided for the first time. After examining these type specimens, an unpublished manuscript name was found and is described herein as *Pterocyclosanamullayensis* Sutcharit & Panha, **sp. n.**

## Introduction

Cyclophoridae Gray, 1847 is a major group of terrestrial operculate snails found across southern Europe, Central America, Asia, Africa, and Australia (Kobelt 1902, Solem 1959, Stanisic 1998, Nantarat et al. 2014a). Many cyclophorid groups are common and widespread, rich in species and are ecologically significant components of tropical habitats. They are both ground and arboreal dwelling, and exhibit a wide range of shell morphology from small (< 5 mm) to large (> 30 mm), turbinate or globose to discoidal. Bouchet and Rocroi (2005) and Bouchet et al. (2017) have recognised four subfamilies, and the nominotypical subfamily consists of five tribes.

The cyclophorinid genera *Pterocyclos* Benson, 1832, *Cyclotus* Swainson, 1840, *Myxostoma* Troschel, 1847, *Rhiostoma* Benson, 1860, *Scabrina* Blanford, 1863, *Crossopoma* Martens, 1891, and *Pearsonia* Kobelt, 1902, represent approximately 180 nominal species, distributed across South and Southeast Asia, southern China and Japan (Kobelt 1902, Marzuki and Clements 2013). Two genera, *Myxostoma* and *Crossopoma*, which comprise only a few species, are considered to be endemic to southern Vietnam and the Sundaic Islands respectively (Henderson 1898, Kobelt 1902, Zilch 1955, Egorov 2009). The genus *Rhiostoma*, endemic to Indochina, consists of approximately fifteen species. In this genus, the last whorl is descending and curved detached-whorl (proboscis-like detached-whorl) and it possesses a calcareous cup-shaped operculum (Kobelt 1902, Egorov 2009). The genera *Pterocyclos*, *Cyclotus*, *Pearsonia*, and *Scabrina* have many similar features; they are discoidal in shape, have a circular aperture (sometimes with distinct accessory breathing device), and a calcareous to corneous operculum. Such similarities have made it difficult not only for species identification but also for generic assignment. The ambiguity in species boundary recognition has led to a limited number of taxonomic revisionary papers at the generic level, but generated numerous standalone species descriptions (i.e., Marzuki and Clements 2013, Sutcharit et al. 2014, Tumpeesuwan and Tumpeesuwan 2015, Foon 2016).

Since the complete morphological revision of these seven genera by Wilhelm Kobelt (1902, 1911–1914), no subsequent works have attempted a systematic rearrangement based on a molecular framework. The few papers dealing with the phylogenetics of Asian cyclophorids show that they are much more genetically diverse than their morphology suggests (Prasankok et al. 2011, Lee et al. 2012, Nantarat et al. 2014b, c, Oheimb et al. 2017). Unlike pulmonate molluscs, the reproductive organs of cycylophorids are more highly conserved and are consequentially less reliable as a taxonomically informative character. Thus, the correct identification of the species based on shell morphology presents a challenge for future phylogenetic approaches.

The traditional classification of *Crossopoma*, *Cyclotus*, *Myxostoma*, *Pearsonia*, *Pterocyclos*, *Rhiostoma* and *Scabrina* dates back to Kobelt (1902, 1911–1914), Gude (1921), Wenz (1938–1944) and Benthem Jutting (1948, 1959), all of which mainly relied on shell shape, accessory breathing device and shell colour pattern. Almost all of the known species have long been described, with only a brief type description, and mostly without illustration or explicitly designation of the name bearing type. Of these recognised species, 59 taxa (mostly described by O Boettger, F Haas, W Kobelt, O Möllendorff, H Rolle, B Rensch, and T-C Yen), whose type specimens are housed in the Forschungsinstitut und Naturmuseum Senckenberg, Frankfurt, are well catalogued and illustrated (Zilch 1955, 1956). Later, Hwang (2014), Raheem et al. (2014), and Sutcharit et al. (2014) have re-investigated and illustrated the type specimens of eleven taxa within seven genera in their respective regional faunistic studies. However, the majority of the remaining taxa have not been investigated or illustrated. The Natural History Museum, London (NHM) collections, is one of the largest museum mollusc collections, with specimens acquired from various sources and collectors (for more information see Dance 1986, Breure and Ablett 2011, Nantarat et al. 2014a, Hwang 2014, Sutcharit et al. 2015). The NHM collections hold the majority of the species described by WT Blanford, H Fulton, HH Godwin-Austen, G Nevill, L Pfeiffer, EA Smith, and GB Sowerby I, amongst others, and in many cases the type specimens have not been catalogued or illustrated since their original publication.

Type specimens provide key species data, as they represent the international standard and form the basis of nomenclatural stability when following the International Commission on Zoological Nomenclature (ICZN) guidelines. Therefore, the objective of this study was to evaluate the type status of *Crossopoma*, *Cyclotus*, *Myxostoma*, *Pearsonia*, *Pterocyclos*, *Rhiostoma*, and *Scabrina* type specimens in the NHM collections and to document and figure them in accordance with ICZN (1999) guidelines.

## Materials and methods

**Collections**: The primary type specimens (i.e., holotype, lectotype, and syntype/s) along with secondary type specimens (paratype/s and paralectotype/s) of *Crossopoma*, *Cyclotus*, *Myxostoma*, *Pearsonia*, *Pterocyclos*, *Rhiostoma*, and *Scabrina* species described from the early of 19^th^ century until the year 2018 are here examined. When considering the type specimens, in publications where a single primary type was not clearly designated, and the available specimens proved to form part of the type series, these are considered to be syntypes. In cases where a holotype was not designated, but it is clear from the original publication that the name was based on a single specimen, these are considered a holotype fixed by monotypy (ICZN 1999: Art. 73.1.2). Lectotypes mentioned in this catalogue are here designated, unless otherwise stated, to enhance the stability of the name.

All specimens considered as forming part of the type series are photographed in the standard positions (apertural, apical, and umbilical views). The original labels have been photographed and checked against the original description. The measurements of the holotype, lectotype, and syntypes were taken in mm with a digital caliper. We have also included specimen data in cases where the primary type is housed in another museum collection but paratype(s) or paralectotype(s) are kept in the NHM collections.

**Presentation**: This illustrated catalogue is listed by current genera with species in alphabetical order, regardless of termination, incorrect original spelling, and the association with the authorships and dates. The synonymy tabulation and the usage of each taxon name have been comprehensively provided in Kobelt (1902, 1911–1914), Gude (1921) and Benthem Jutting (1948, 1959). The original combination of the taxon name with reference to pages, plate, and/or figures that made the names available is mentioned. In addition, we also list references where type specimens have been subsequently mentioned or illustrated, especially publications like the “*Conchologia Iconica*…” by Reeve (1861, 1862, 1863), “*Systematisches Conchylien-Cabinet*…” by Pfeiffer (1849, 1853b, 1854a) and “*Conchologia Indica*…” by Hanley and Theobald (1870–1876). The type locality is as stated in the original publication in the original wording and language. Additional locality data from original labels, with respect to current political boundaries or subsequently published localities is given in square brackets. Under the type materials, primary type specimens with the Natural History Museum registration number (hereafter NHMUK), number of specimen(s), and the figures as listed in this publication are given. In addition, if the paratype(s) of that taxa are present; the registration number, number of specimens, and figures of representative specimen are also given. The history and type evidence is summarised under each taxon. Full bibliographic references are provided at the end of this paper.

**Institutional abbreviations**: Abbreviation of the museum collection appeared below in the lists of taxa and species descriptions are listed as follows:

**ANSP** Academy of Natural Sciences of Philadelphia, Drexel University, Philadelphia

**CUMZ** Chulalongkorn University, Museum of Zoology, Bangkok

**NHMUK** Natural History Museum, London

**NMW** The National Museum of Wales, Cardiff

**RBINS** Royal Belgian Institute of Natural Sciences, Brussels

**RMNH** Rijksmuseum van Natuurlijke Historie, Leiden

**SMF** Forschungsinstitut und Naturmuseum Senckenberg, Frankfurt am Main

## Results

There are 95 available taxa that are classified into the seven genera of *Cyclotus*, *Crossopoma*, *Myxostoma*, *Scabrina*, *Personia*, *Pterocyclos* and *Rhiostoma*. Twelve species names “*atronitens*”, “*burrailensis*”, “*butleri*”, “*coorgensis*”, “*daflensis*”, “*hengdanensis*”, “*hirtus*”, “*lahupaensis*”, “*lemani*”, “*lhotaensis*”, “*parrus*”, and “*sylhetensis*” are unpublished and found only on the labels of the specimens in Godwin-Austen collection, which are considered as unavailable nominal taxa (ICZN 1999: Art. 12). The un-published taxon name “*Pterocyclos anamullayensis*” in the Beddome collection is clearly distinct from all other known species, and is described here as a new species. Amongst these available taxa, the NHM retained 96 % of the name-bearing types exclusively as 12 holotypes, 15 lectotypes, and 64 lots of syntype material. In the case of the five holotype lots of “*bathyrhaphe*”, “*brounae*”, “*gwendolenae*”, “*hungerfordi*”, and “*spiramentum*”, the type status has only recently been confirmed and is recognized as the holotype (fixed by monotypy). The five probable/possible syntype lots are “*cochinchinensis*”, “*inglisianus*”, “*politus*”, “*puriensis*”, and “*volvuloides*”. Among the 15 lectotype lots, eleven lots are here designated as the lectotypes to clarify their type status and promote the stability of the taxon name. The other four lots were previously designated from the original type series by Raheem et al. (2014) as “*comatus*”, “*cumingi*”, and “*fairbanksi*”, and by Hwang (2014) as “*taivanus*”. The remaining 4 % are solely paratypes, whose name-bearing types are housed elsewhere.

### Catalogue of type material

#### 1. abletti

Taxon classificationAnimaliaArchitaenioglossaCyclophoridae

Thach, 2016

Fig. 1A

Rhiostoma abletti Thach, 2016: 37, 38, figs 53, 122–124.

##### Current generic position.

*Rhiostoma* Benson, 1860

##### Type locality.

Northwest of Lai Châu city, on the way going to Paso, Lai Châu Province (north Vietnam).

##### Type material.

**Figure 1.**
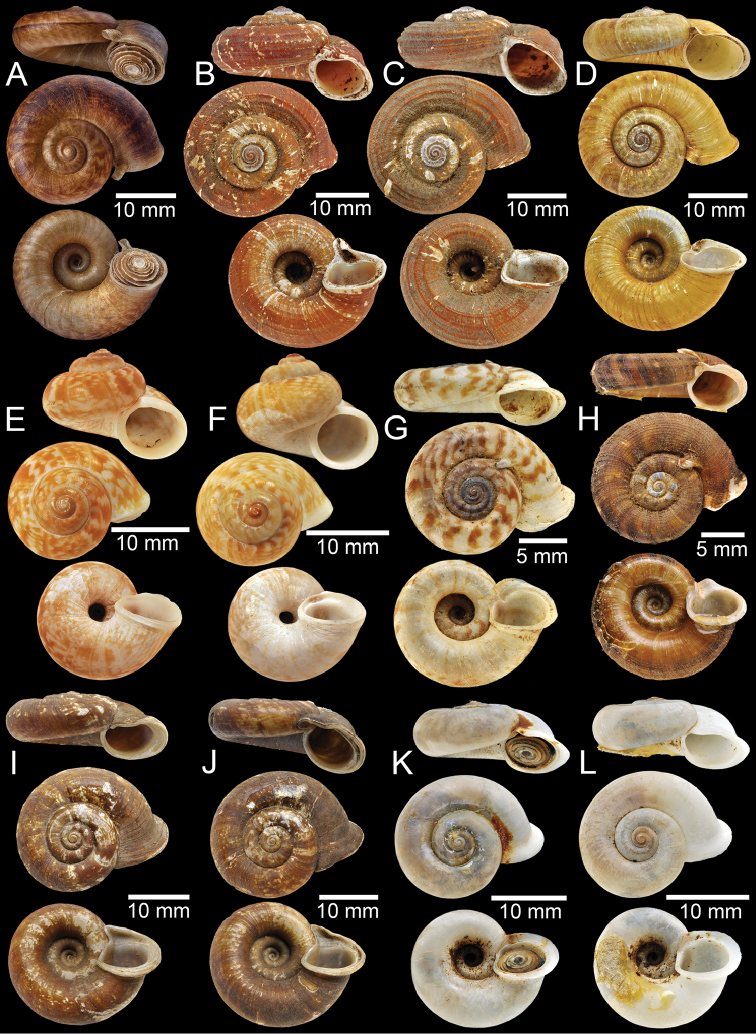
**A** Holotype of *Rhiostomaabletti***B, C** syntype of *Pterocyclosaborensis***D** syntype of *Cyclotusamabilis***E, F***Cyclotusamboinensis***E** lectotype and **F** paralectotype **G, H** syntype of *Pearsoniaandersoni***I, J** syntype of *Pterocyclosanguliferus***K, L** syntype of *Pterocyclosaspesrus*.

Holotype NHMUK 20160307 (Fig. 1A), paratype ANSP 467381 (1 shell).

#### 2. aborensis

Taxon classificationAnimaliaArchitaenioglossaCyclophoridae

Godwin-Austen, 1915

Fig. 1B, C

Pterocyclos aborensis Godwin-Austen, 1915: 498, pl. 39, figs 1, 1a. Gude 1921: 98.

##### Current generic position.

*Pterocyclos* Benson, 1832

##### Type locality.

Abor Hills; Pongping; Rami Lampang [region in Arunachal Pradesh State, India].

##### Type material.

Syntype NHMUK 1903.7.1.3104 (3 shells; Fig. 1B, C) from Abor Hills; NHMUK 1903.7.1.3046 (2 shells) from Pongping, Abor; NHMUK 1903.7.1.3050 (3 shells) from Rami Lambang, Abor.

##### Remarks.

Godwin-Austen’s description was based on three lots of specimens. The original description includes an illustration and one set of measurements. The original description stated “Type no. 3104 Brit. Mus.” There are three specimens from Godwin-Austen type collection that relate to this registration number. The specimen figured in the original description which corresponds to the measurements given is figured herein (Fig. 1B).

#### 3. amabilis

Taxon classificationAnimaliaArchitaenioglossaCyclophoridae

Fulton, 1905

Fig. 1D

Cyclotus (Eucyclotus) amabilis Fulton, 1905: 93.

##### Current generic position.

*Cyclotus* Swainson, 1840

##### Type locality.

N. Borneo [North Borneo].

##### Type material.

Syntype NHMUK 1905.4.14.4 (1 shell; Fig. 1D).

##### Remarks.

The original description did not include an illustration and only one set of shell measurements was given. The author stated “sometime zigzag”, which implied that this taxon was based on more than one specimen. The NHM collections contain a lot comprised of a single specimen purchased from Sowerby and Fulton with a label stating “Type” and with a collection locality written on the original label, this specimen is figured herein.

#### 4. amboinensis

Taxon classificationAnimaliaArchitaenioglossaCyclophoridae

(Pfeiffer, 1854)

Fig. 1E, F

Cyclostoma (Cyclophorus) amboinense Pfeiffer, 1854b [1852]: 144. Pfeiffer 1854a: 373, pl. 48, figs 20–22.
Cyclotus amboinensis — Kobelt 1902: 189.

##### Current generic position.

*Cyclotus* Swainson, 1840

##### Type locality.

Amboyna [Ambon Island, Maluku Province, Indonesia].

##### Type material.

Lectotype (design. n.) NHMUK 20170352/1 (Fig. 1E), paralectotypes NHMUK 20170352/2–3 (2 shells; Fig. 1F).

##### Remarks.

The original description lacked mention of an operculum and did not include an illustration, and only one set of shell measurements was given. Pfeiffer (1854a) re-published the description and figured the species which no operculum. There is a lot of four shells from the Cuming collection with two Pfeiffer handwritten labels. The larger label states “*C. Amboinensis* Pfr.”, “The operculum shows that…” and the collection locality from “Isle of Lobok”. We presume that this lot contains a subsequently introduced specimen that has an operculum. Therefore, the specimen with an operculum attached (NHMUK 20070352/4) is excluded from the type series. The remaining three shells have no operculum and the smaller label states “*Cycl. Amboinense* Pfr.” and gives the collection locality as “Amboyana”. The specimen that most closely matches with the measurements given in the original description and the illustration in Pfeiffer (1854a) is here designated as the lectotype to stabilise the name.

#### 5. andersoni

Taxon classificationAnimaliaArchitaenioglossaCyclophoridae

(Blanford, 1869)

Fig. 1G, H

Spiraculum andersoni Blanford, 1869b: 447.
Pterocyclos andersoni — Hanley and Theobald 1872: 23, pl. 49, figs 3, 4.
Pearsonia andersoni — Kobelt 1902: 172. Kobelt 1911: 766, 767, pl. 112, figs 10, 11.

##### Current generic position.

*Pearsonia* Kobelt, 1902

##### Type locality.

ad Bhamo cum praecedente [Bhamo District, Kachin State, Myanmar].

##### Type material.

Syntype NHMUK 1906.5.5.77 (3 shells; Fig. 1G, H).

##### Remarks.

The species description was clearly based on more than one specimen, but an illustration was not included in the original description. Later, the species was figured in Hanley and Theobald (1872). The figured specimen with red wool inside the aperture is illustrated in Figure 1G.

#### 6. anguliferus

Taxon classificationAnimaliaArchitaenioglossaCyclophoridae

(Souleyet, 1841)

Fig. 1I, J

Cyclostoma angulifera Souleyet, 1841: 347. Eydoux and Souleyet 1852: 530–532, pl. 30, figs 6–11.
Pterocyclos anguliferus — Reeve 1863: volume 14, Pterocyclos, pl. 2, species 10. Kobelt 1902: 161.

##### Current generic position.

*Pterocyclos* Benson, 1832

##### Type locality.

Les environs de Touranne (Cochinchine) [Da Nang City, Vietnam].

##### Type material.

Syntype NHMUK 1854.7.24.365 (2 shells; Fig. 1I, J).

##### Remarks.

The original description included one set of measurements. Later, Eydoux and Souleyet (1852) re-published the description and figured this species. Gray (1855: 19) made a list of type specimens of molluscs described by the late M Souleyet and housed in the NHM collections. There are two specimens with an original label stating the species name and collection locality. The specimen that closely matches the figure in Eydoux and Souleyet (1852) and is closest to the measurements given in the original description is illustrated in Figure 1I.

#### 7. aspersus

Taxon classificationAnimaliaArchitaenioglossaCyclophoridae

Bullen, 1906

Fig. 1K, L

Pterocyclos aspersus Bullen, 1906: 129, with text figure. Kobelt 1911: 752, 753, pl. 111, figs 11–13a.

##### Current generic position.

*Pterocyclos* Benson, 1832

##### Type locality.

Loeboek, Bangko [region in Bangko, Merangin Regency, Jambi Province, Indonesia].

##### Type material.

Syntype NHMUK 1906.7.21.1 (5 adults + 1 juvenile; Fig. 1K, L).

##### Remarks.

The original description is clearly based on more than one specimen, but only one set of measurements and illustrations were given. The NHM type collections contain a lot of six shells with the label stating the taxon name, collection locality, and “Type”. The specimen figured in the original description, which is closest to the shell dimensions given in the original description, is illustrated in Figure 1K.

#### 8. assamenseis

Taxon classificationAnimaliaArchitaenioglossaCyclophoridae

(Fulton, 1900)

Fig. 2A, B

Spiraculum assamense Fulton, 1900: 87, 88.
Pearsonia assamensis — Gude 1921: 113, 114, fig. 18.

##### Current generic position.

*Pearsonia* Kobelt, 1902

##### Type locality.

Khasi Hills, Assam [Khasi Hills, Meghalaya State, India].

##### Type material.

**Figure 2.**
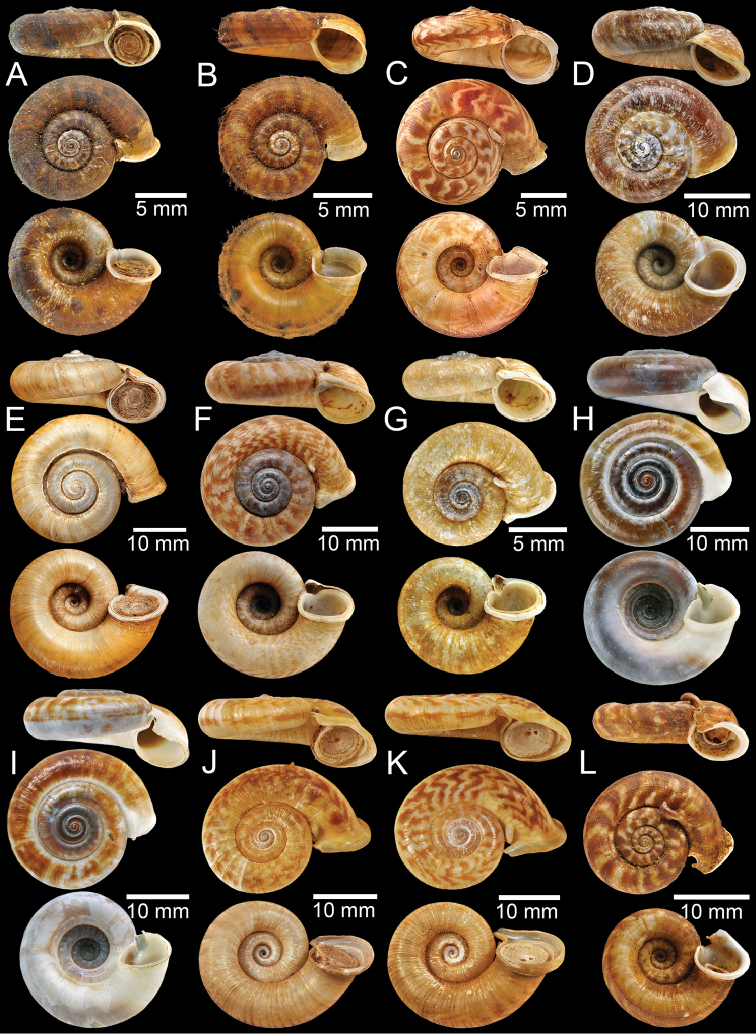
**A, B** Syntype of *Pearsoniaassamenseis***C** syntype of *Pearsoniaavana***D** syntype of *Cyclotusbatchianensis***E** holotype of *Crossopomabathyrhaphe***F** syntype of *Pearsoniabeddomei***G** syntype of *Pearsoniabhamoensis***H, I** syntype of *Pterocyclosbifrons***J, K** syntype of *Cyclotusbirostris***L** syntype of *Pearsoniabitubifera*.

Syntype NHMUK 1901.4.25.45–46 (2 shells; Fig. 2A, B).

##### Remarks.

The original description did not include an illustration, and only one set of shell measurements was given. There are two shells in the NHM collection with Fulton’s handwritten label stating “TYPE (larger)”. The larger specimen corresponds to the measurements given in the original description and is illustrated in Figure 2A.

#### 9. avana

Taxon classificationAnimaliaArchitaenioglossaCyclophoridae

(Blanford, 1863)

Fig. 2C

Spiraculum avanum Blanford, 1863: 319–321.
Pterocyclos avanus — Hanley and Theobald 1875: 54, pl. 134, figs 8, 9.
Pearsonia avana — Kobelt 1902: 172.

##### Current generic position.

*Pearsonia* Kobelt, 1902

##### Type locality.

Shan Hills, east of the town of Ava [Shan Hills, Kyaukse District, Mandalay Region, Myanmar].

##### Type material.

Syntype NHMUK 1903.7.1.4198 (1 shell; Fig. 2C).

##### Remarks.

The species was clearly based on two specimens, one dead and one alive. The original description did not include an illustration and only one set of measurements was given. Subsequently, Hanley and Theobald (1875) figured a specimen of this species. The single specimen in the NHM from the Godwin-Austen collection, ex. WT Blanford collection and figured in Hanley and Theobald (1875) is figured herein.

#### 10. batchianensis

Taxon classificationAnimaliaArchitaenioglossaCyclophoridae

Pfeiffer, 1861

Fig. 2D

Cyclotus batchianensis Pfeiffer, 1861: 28, pl. 3, fig. 1. Kobelt 1902: 197.
Pterocylos batchianensis — Reeve 1863: volume 14, Pterocyclos, pl. 2, species 6.

##### Current generic position.

*Cyclotus* Swainson, 1840

##### Type locality.

Ise of Batchian [Bacan Islands, north Maluku Province, Indonesia].

##### Type material.

Syntype NHMUK 20170364 (1 adult + 1 juvenile; Fig. 2D).

##### Remarks.

The original description by Pfeiffer includes an illustration and one set of shell measurements. The type lot in the NHM collections was collected by “Mr. Wallace” and is from the Cuming collection as stated in the original description. It has an original label in Pfeiffer’s handwritings giving the species name and collection locality. The adult specimen that closely matches the measurements and the illustration shown in the original description is figured herein.

#### 11. bathyrhaphe

Taxon classificationAnimaliaArchitaenioglossaCyclophoridae

(Smith, 1878)

Fig. 2E

Cyclophorus (Myxostoma) bathyrhaphe Smith, 1878: 497–499, fig. 3.
Crossopoma bathyrhaphe — Kobelt 1902: 85.

##### Current generic position.

*Crossopoma* Martens, 1891

##### Type locality.

Borneo.

##### Type material.

Holotype NHMUK 1878.1.30.1 (Fig. 2E).

##### Remarks.

Smith clearly stated that this taxon was described based on a single specimen from the GB Sowerby I collection. The species description included an illustration and a set of shell dimensions. The NHM collections contain a type lot that has an original label stating “Type”, subsequently re-written as “Holotype”. Therefore, we recognise this single shell as the holotype fixed by monotypy.

#### 12. beddomei

Taxon classificationAnimaliaArchitaenioglossaCyclophoridae

(Blanford, 1866)

Fig. 2F

Spiraculum beddomei Blanford, 1866: 31, 32.
Pterocyclos beddomei — Hanley and Theobald 1875: 54, pl. 134, figs 5, 6.
Pearsonia beddomei — Kobelt 1902: 172, 173, fig. 36. Kobelt 1911: 767, 768, pl. 112, figs 14–18.

##### Current generic position.

*Pearsonia* Kobelt, 1902

##### Type locality.

Kimeky Hills near Waltair (Vizagapatam), northern division of the Madras Presidency [Visakhapatnam District, Andhra Pradesh State, India].

##### Type material.

Syntype NHMUK 1906.1.1.942 (1 shell; Fig. 2F).

##### Remarks.

The species description was based on more than one specimen. The original description did not include an illustration, and only one set of measurements was given. Hanley and Theobald (1875) subsequently figured this species. There is a specimen in the NHM from the Blanford collection figured in Hanley and Theobald (1875) that closely matches the dimensions given in the original description, it is figured herein.

#### 13. bhamoensis

Taxon classificationAnimaliaArchitaenioglossaCyclophoridae

(Theobald, 1876)

Fig. 2G

Spiraculum bhamoense Theobald, 1876: 186, 187.
Pearsonia bhamoensis — Kobelt 1902: 173.

##### Current generic position.

*Pearsonia* Kobelt, 1902

##### Type locality.

Bhamo valle Iravadi Regno Burmanico [Ayeyarwady Valley, Bhamo District, Kachin State, Myanmar].

##### Type material.

Syntype NHMUK 1888.12.4.1964 (1 shell; Fig. 2G).

##### Remarks.

The original description did not include an illustration, and only one set of measurements was given. Gude (1921: 116, 117, fig. 20) re-published the description and figured a type specimen from Theobald’s collection. There is one shell in the NHM collection purchased from W Theobald, with an original label stating “type” and the collection locality “Bhamo”. The specimen figured herein closely matches the measurements given in the original description.

#### 14. bifrons

Taxon classificationAnimaliaArchitaenioglossaCyclophoridae

Pfeiffer, 1855

Fig. 2H, I

Pterocyclos bifrons Pfeiffer, 1855b: 117. Reeve 1863: volume 14, Pterocyclos, pl. 1, species 1. Kobelt 1902: 162, 163.

##### Current generic position.

*Pterocyclos* Benson, 1832

##### Type locality.

Ceylon [Sri Lanka].

##### Type material.

Syntype NHMUK 20170365 (3 shells; Fig. 2H, I).

##### Remarks.

The species was described based on specimens from the Cuming collection. The original description did not include illustrations, and only one set of measurements was given. Reeve (1863) re-described and illustrated a single specimen from the Cuming collection. There are three specimens from the Cuming collection in the NHM type lot with an original label in Pfeiffer’s handwriting stating the species name and collection locality. The specimen figured in Reeve (1863) closely matches the measurements given in the original description is illustrated here in Figure 2H.

#### 15. birostris

Taxon classificationAnimaliaArchitaenioglossaCyclophoridae

(Pfeiffer, 1855)

Fig. 2J, K

Cyclostoma birostre Pfeiffer, 1855a [1854]: 300.
Pterocyclos birostris — Reeve 1863: volume 14, Pterocyclos, pl. 4, species 18.
Cyclotus birostris — Kobelt 1902: 214.

##### Current generic position.

*Cyclotus* Swainson, 1840

##### Type locality.

Sarawak, Borneo [Sarawak, Malaysia].

##### Type material.

Syntype NHMUK 20170353 (3 shells; Fig. 2J, K).

##### Remarks.

This species was described based on material from the Cuming collection, and only one set of shell measurements was given. Later, Reeve (1863) re-described the species and illustrated a shell from the Cuming collection. There are three specimens in the NHM collections with an original label stating “Rev C. I. f. 18a–b”. The specimen figured herein (Fig. 2J) is closest to the illustration in Reeve (1863) and the shell dimensions given in the original description.

#### 16. bitubifera

Taxon classificationAnimaliaArchitaenioglossaCyclophoridae

(Theobald, 1876)

Fig. 2L

Spiraculum bitubiferum Theobald, 1876: 187.
Pearsonia bitubifera — Kobelt 1902: 173.

##### Current generic position.

*Pearsonia* Kobelt, 1902

##### Type locality.

Bhamo [Bhamo District, Kachin State, Myanmar].

##### Type material.

Syntype NHMUK 1888.12.4.1961 (1 shell; Fig. 2L).

##### Remarks.

The original description did not include an illustration, and only one set of measurements was given. Gude (1921: 117, 118, fig. 21) re-published the description and figured a shell from Theobald’s collection. There is one shell in the NHM collection purchased from W Theobald, with an original label stating “type” and the collection locality “Bhamo”. This specimen closely matches the measurements given in the original description and is figured herein.

#### 17. boxalli

Taxon classificationAnimaliaArchitaenioglossaCyclophoridae

Godwin-Austen, 1889

Fig. 3A

Cyclotus boxalli Godwin-Austen, 1889: 343, 344, pl. 36, fig. 4, 4a. Kobelt 1902: 212.

##### Current generic position.

*Cyclotus* Swainson, 1840

##### Type locality.

Molu Hills [Gunung Mulu National Park, Miri Division, Sarawak, Malaysia].

##### Type material.

**Figure 3.**
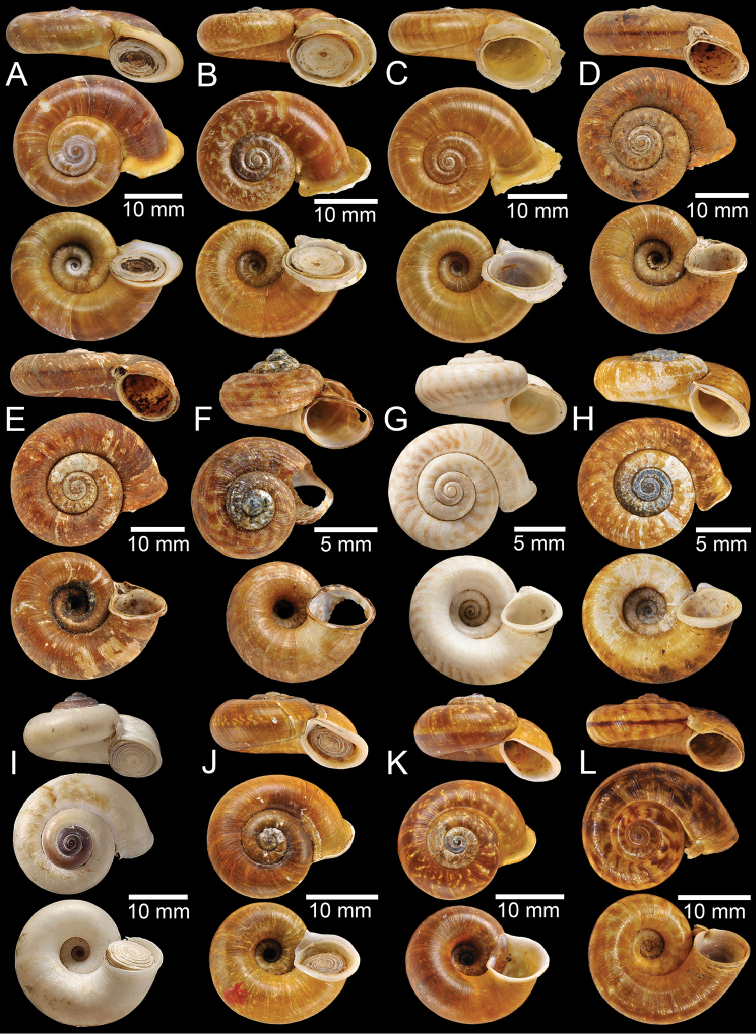
**A** Syntype of *Cyclotusboxalli***B, C** syntype of *Rhiostomaboxalli***D, E** syntype of *Pterocyclosbrahmakundensis***F** holotype of *Scabrinabrounae***G, H** syntype of *Scabrinacalyx***I** syntype of *Rhiostomacambodjense***J, K** syntype of *Pterocycloscelebensis***L** holotype of *Rhiostomachristae*.

Syntype NHMUK 1891.3.17.35 (1 shell; Fig. 3A).

##### Remarks.

This species was described from the Hungerford collection. An illustration and a set of shell dimensions were given in the original description. Godwin-Austen (1889) does not explicitly indicate the number of specimens he has available to him in the original description. There is a single shell in the NHM collections from the R Hungerford collection with an original label stating “Type”, the species name, and collection locality. This specimen closely matches with the illustration and the shell measurements given in the original description, and it is figured herein.

#### 18. boxalli

Taxon classificationAnimaliaArchitaenioglossaCyclophoridae

Godwin-Austen, 1893

Fig. 3B, C

Rhiostoma boxalli Godwin-Austen, 1893: 32, 33, fig. 1a–c. Kobelt 1902: 538, 539.

##### Current generic position.

*Rhiostoma* Benson, 1860

##### Type locality.

Near Kina Balu [Mount Kinabalu, Sabah, Malaysia]; Palawan [Palawan Islands, Province of Palawan, Mimaropa Region, Philippines].

##### Type material.

Syntypes NHMUK 1894.5.23.1 from near Kina Balu (1 shell; Fig. 3B), NHMUK 1895.12.5.34 from Palawan (1 shell; Fig. 3C).

##### Remarks.

The original description gives a set of shell measurements and figures of two specimens, so this species is clearly based on more than one specimen. There is a type lot (two shells) with differing registration numbers, one specimen NHMUK 1894.5.23.1 is from R Hungerford ex. Mr. Boxall from Kina Balu and the NHM registration book states “Type”. The other shell, NHMUK 1895.12.5.34, in the same box was collected by Mr. Whitehead from Palawan and agrees well with Godwin-Austen (1893: fig. 1b, c). These two shells form part of the type series and are considered as syntypes.

#### 19. brahmakundensis

Taxon classificationAnimaliaArchitaenioglossaCyclophoridae

Godwin-Austen, 1915

Fig. 3D, E

Pterocyclos brahmakundensis Godwin-Austen, 1915: 499, 500, with text figure 1. Gude 1921: 101.

##### Current generic position.

*Pterocyclos* Benson, 1832

##### Type locality.

Brahmakund, eastern Assam [Parshuram Kund, Lohit District, Arunachal Pradesh State, India].

##### Type material.

Syntype NHMUK 1903.7.1.713 (3 shells; Fig. 3D, E).

##### Remarks.

Godwin-Austen clearly stated that the original description was based on a lot of three shells “Type No. 713 B.M.”. The NHM type collections contain a lot of three shells from the Godwin-Austen collection and original label states “TYPE”. The specimen with red wool inside the aperture that corresponds to the illustrations and measurements given in the original description, and it is figured herein (Fig. 3D).

#### 20. brounae

Taxon classificationAnimaliaArchitaenioglossaCyclophoridae

(Sykes, 1898)

Fig. 3F

Cyclophorus (Scabrinus) brounae Sykes, 1898: 73, figs 2, 3.
Scabrina brounae — Kobelt 1902: 78.

##### Current generic position.

*Scabrina* Blanford, 1863

##### Type locality.

Nuwara–Eliya [Nuwara Eliya District, Central Province, Sri Lanka].

##### Type material.

Holotype NHMUK 1903.7.17.3 (Fig. 3F).

##### Remarks.

Sykes clearly stated that this taxon was described based on a single specimen collected by Mrs. Broun. The original description included an illustration and a set of shell measurements. The NHM collections contain a Sykes type lot that has an original label stating “Type”, and so we recognise this single illustrated specimen as the holotype fixed by monotypy.

#### 21. calyx

Taxon classificationAnimaliaArchitaenioglossaCyclophoridae

(Benson, 1856)

Fig. 3G, H

Cyclophorus calyx Benson, 1856: 228, 229. Pfeiffer 1860b: 145, 146, pl. 37, figs 25–27. Reeve 1861: volume 13, Cyclophorus, pl. 20, species 104.
Scabrina calyx — Kobelt 1902: 87, 88.

##### Current generic position.

*Scabrina* Blanford, 1863

##### Type locality.

ad Akaouktong, prope ripas fluminis Irawadi [Akauk Taung (Hill), Padaung Township, Pyay District, Bago Region, Myanmar].

##### Type material.

Syntype NHMUK 1954.6.2.1542–1544 (3 shells; Fig. 3G, H).

##### Remarks.

The original description did not include an illustration or state the number of specimens examined and there is no information concerning the operculum, however one set of shell measurements were given. Later, Pfeiffer (1860b) and Reeve (1861) re-published the description and figured the species from specimens in the Benson collection. There is a type lot in the NHM ex. Hawkins collection containing three shells (1 with and 2 without periostracum). The specimen without periostracum that most closely matches with the measurements given in the original description and the illustrations in Pfeiffer (1860b: pl. 37, figs 25–27) and Reeve (1861: pl. 20, species 104) is figured herein (Fig. 3G).

#### 22. cambodjense

Taxon classificationAnimaliaArchitaenioglossaCyclophoridae

(Morelet, 1875)

Fig. 3I

Pterocyclos cambodjensis Morelet, 1875: 286, 287, pl. 13, fig. 1. Breure et al. 2018: 232, figs 173, 174.
Rhiostoma cambodjense — Kobelt 1902: 177. Kobelt 1911: 763, 764, pl. 113, figs 14–16.

##### Current generic position.

*Rhiostoma* Benson, 1860

##### Type locality.

Battambang, Cambodje [Battambang Province, Cambodia].

##### Type material.

Syntype NHMUK 1893.2.4.766 (1 shell; Fig. 3I).

##### Remarks.

The original description includes an illustration and one set of shell measurements is given. However, the species description was not clearly based on a single specimen. Only one shell was found in the NHM collections with an original label stating “Type” and giving the reference of the original description and collection locality. This figured specimen exactly matches with the illustration and shell measurements given in the original description.

#### 23. celebensis

Taxon classificationAnimaliaArchitaenioglossaCyclophoridae

(Smith, 1896)

Fig. 3J, K

Cyclotus celebensis Smith, 1896b: 101, pl. 7, figs 4–6.
Pterocyclos celebensis — Kobelt 1902: 163, 164.

##### Current generic position.

*Pterocyclos* Benson, 1832

##### Type locality.

South Celebes, 2000–4000 feet [South Sulawesi Province, Indonesia].

##### Type material.

Syntype NHMUK 1896.5.1.3 (2 shells; Fig. 3J, K).

##### Remarks.

There are two specimens in the type lot with original labels in Smith’s handwriting stating “Type”. The original description gives one set of measurements and illustrates one specimen. The specimen that corresponds to the illustrations in Smith (1896b: figs 4–6) and the given shell measurements in the original description and has a red spot on the last whorl is figured herein (Fig. 3J).

#### 24. chinensis

Taxon classificationAnimaliaArchitaenioglossaCyclophoridae

(Pfeiffer, 1855)

Fig. 4A, B

Cyclostoma (Cyclotus) chinense Pfeiffer, 1855a [1854]: 299.
Leptopoma chinense — Reeve 1862: volume 13, Leptopoma, pl. 7, species 43.
Cyclotus chinensis — Kobelt 1902: 205.

##### Current generic position.

*Cyclotus* Swainson, 1840

##### Type locality.

Hong Kong, China.

##### Type material.

**Figure 4.**
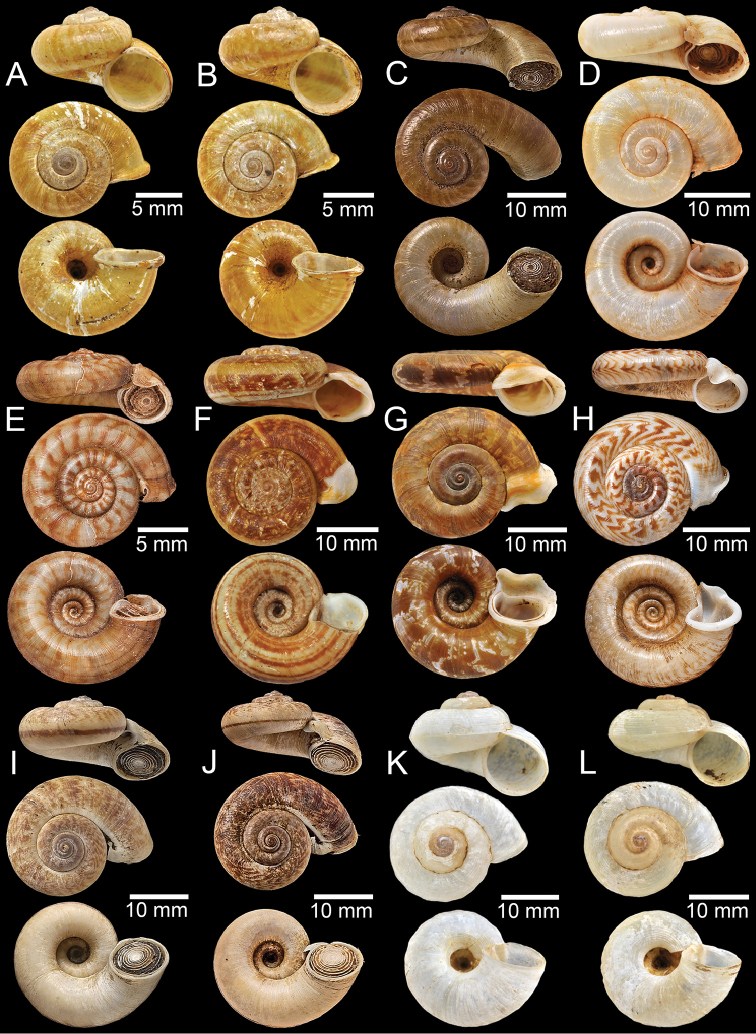
**A, B** Syntype of *Cyclotuschinensis***C** lectotype of *Rhiostomachupingense***D** probable syntype of *Cyclotuscochinchinensis***E** lectotype of *Pterocycloscomatus***F** syntype of *Cyclotusconfluens***G** syntype of *Pterocycloscucullus***H** lectotype of *Pterocycloscumingi***I, J** syntype of *Rhiostomadalyi***K, L** syntype of *Cyclotusdaucinus*.

Syntype NHMUK 198040 (3 shells; Fig. 4A, B).

##### Remarks.

The original description did not include an illustration, and one set of shell measurements was given. Later, Reeve (1862) re-published the description and figured a shell from the Cuming collection. There is one lot from the Cuming collection containing three specimens collected by Mr. Fortune with an original label in Pfeiffer’s handwriting stating the species name and collection locality. The specimen that most closely matches the measurements given in the original description and the illustration in Reeve (1862) is figured herein (Fig. 4A).

#### 25. christae

Taxon classificationAnimaliaArchitaenioglossaCyclophoridae

Thach, 2016

Fig. 3L

Rhiostoma christae Thach, 2016: 38, figs 51, 130–133.

##### Current generic position.

*Rhiostoma* Benson, 1860

##### Type locality.

Near the road No. 6 to Chieng Ngan, Son La Province (north Vietnam).

##### Type material.

Holotype NHMUK 20160306 (Fig. 3L), paratype ANSP 467386 (1 shell).

#### 26. chupingense

Taxon classificationAnimaliaArchitaenioglossaCyclophoridae

Tomlin, 1938

Fig. 4C

Rhiostoma chupingense Tomlin, 1938: 73, p1. 2, figs 1, 2.

##### Current generic position.

*Rhiostoma* Benson, 1860

##### Type locality.

Bukit Chuping, Perlis, Malaysia.

##### Type material.

Lectotype (design. n.) NHMUK 1938.10.25.1 (Fig. 4C), paralectotypes NMW 1955.158.01101 (3 shells) and NMW.Z 1981.118.02703 (1 shell).

##### Remarks.

The original description was clearly based on more than one specimen, and included an illustration and one set of shell measurements. There is only one specimen in the NHM type collections with Tomlin’s hand written label stating “Type” and the collection locality. This specimen exactly matches with the illustration (not full adult stage) and the measurements given in the original description and is here designated as the lectotype to stabilise the name. The other two lots of Tomlin collections in the NMW with the label stating “paratype” are therefore considered as paralectotypes.

#### 27. cochinchinensis

Taxon classificationAnimaliaArchitaenioglossaCyclophoridae

(Pfeiffer, 1857)

Fig. 4D

Cyclostoma (Opisthophorus) cochinchinense Pfeiffer, 1857a [1856]: 337.
Pterocyclos cochinchinensis — Reeve 1863: volume 14, Pterocyclos, pl. 4, species 22.
Cyclotus cochinchinensis — Kobelt 1902: 209.

##### Current generic position.

*Cyclotus* Swainson, 1840

##### Type locality.

Cochinchina [south of Vietnam].

##### Type material.

Probable syntype NHMUK 20170354 (1 shell; Fig. 4D).

##### Remarks.

This species was described from specimens in the Cuming collection and only one set of shell measurements was given in the original description. The NHM collections contains a lot of a single shell from the Cuming collection with an original label, probably in Pfeiffer’s handwriting, stating the species name, however this has subsequently been overwritten. Reeve (1863) illustrated a single specimen from the Cuming collection. This single specimen closely matches the illustration in Reeve (1863) but is slightly larger than the shell dimensions given in the original description. Therefore, we consider this specimen to be a probable syntype.

#### 28. comatus

Taxon classificationAnimaliaArchitaenioglossaCyclophoridae

Beddome, 1881

Fig. 4E

Pterocyclus comatus Beddome in Nevill, 1881: 146. Kobelt 1902: 164, 165.
Pterocyclos comatus — Raheem et al. 2014: 42, fig. 23d, e.

##### Current generic position.

*Pterocyclos* Benson, 1832

##### Type locality.

Anaamullays.

##### Type specimen.

Lectotype (designated by Raheem et al. 2014), NHMUK 1912.04. 16.669/1 (Fig. 4E), paralectotypes NHMUK 1912.04.16.669/2 (2 shells), SMF 130504 (4 shells).

##### Remarks.

One lot of four specimens SMF 130504/4 in Möllendorff ex. Beddome collection from “Anamullay–Berge” and labelled “Cotypen” are considered as paralectotypes.

#### 29. confluens

Taxon classificationAnimaliaArchitaenioglossaCyclophoridae

(Pfeiffer, 1860)

Fig. 4F

Cyclophorus confluens Pfeiffer, 1860a: 140. Reeve 1861: volume 13, Cyclophorus, pl. 15, species 69.
Japonia (Lagochilus) confluens — Kobelt 1902: 40.

##### Current generic position.

*Cyclotus* Swainson, 1840

##### Type locality.

Borneo.

##### Type material.

Syntype NHMUK 20170355 (1 shell; Fig. 4F).

##### Remarks.

The species description by Pfeiffer did not indicate the number of available specimens and did not include an illustration. Only one set of shell measurements was given in the original description. Reeve (1861) re-published the description and figured one specimen from the Cuming collection. The NHM collections contain a lot containing one shell with Pfeiffer’s handwritten label giving the species name and collection locality. This figured shell matches well with the measurements given in the original description and the figure in Reeve (1861).

#### 30. cucullus

Taxon classificationAnimaliaArchitaenioglossaCyclophoridae

Godwin-Austen, 1889

Fig. 4G

Pterocyclos cucullus Godwin-Austen, 1889: 340, pl. 35, figs 2, 2a. Kobelt 1902: 165.

##### Current generic position.

*Pterocyclos* Benson, 1832

##### Type locality.

Niah Hills [Niah National Park, Miri Division, Srawak, Malaysia].

##### Type material.

Syntype NHMUK 1889.12.7.17 (1 shell; Fig. 4G).

##### Remarks.

The original description included an illustration and one set of shell measurements. Only one specimen is housed in the NHM type collections, with an original label stating “Type”. This shell closely matches the illustration and the measurements given in the original description.

#### 31. cumingi

Taxon classificationAnimaliaArchitaenioglossaCyclophoridae

Pfeiffer, 1851

Fig. 4H

Pterocyclos cumingi Pfeiffer, 1851: 5. Kobelt 1902: 165. Reeve 1863: volume 14, Pterocyclos, pl. 3, species 14. Raheem et al. 2014: 43, figs 23f, 24a.

##### Current generic position.

*Pterocyclos* Benson, 1832

##### Type locality.

insula Ceylon [Sri Lanka].

##### Type material.

Lectotype (designated by Raheem et al. 2014) NHMUK 20110220/1 (Fig. 4H).

#### 32. dalyi

Taxon classificationAnimaliaArchitaenioglossaCyclophoridae

Blanford, 1902

Fig. 4I, J

Rhiostoma dalyi Blanford, 1902: 34, 35, fig. 1.

##### Current generic position.

*Rhiostoma* Benson, 1860

##### Type locality.

Juxta Phitsanulok, in sylvis humidis et densis [Phitsanulok Province, Thailand].

##### Type material.

Syntype NHMUK 1902.1.24.14–16 (3 shells; Fig. 4I, J).

##### Remarks.

This species was described from specimens collected by Mr. WM. Daly from Thailand. The original description included an illustration and a set of shell dimensions. The NHM collections contain a lot of three specimens collected by Mr. Daly and have an original label stating the species name and collection locality. The specimen which is identical to the illustration, particularly in respect to the breathing device, and the shell measurements given in the original description is figured herein (Fig. 4I).

#### 33. daucinus

Taxon classificationAnimaliaArchitaenioglossaCyclophoridae

(Pfeiffer, 1857)

Fig. 4K, L

Cyclostoma (Cyclotus) daucinum Pfeiffer, 1857a [1856]: 337.
Cyclotus daucinus — Reeve 1863: volume 14, Cyclotus, pl. 8, species 49.

##### Current generic position.

*Cyclotus* Swainson, 1840

##### Type locality.

Salomon’s Islands [Solomon Islands].

##### Type material.

Syntype NHMUK 20170356 (2 shells; Fig. 4K, L).

##### Remarks.

This species was described from material in the Cuming collection, and the original description included only one set of shell dimensions. Reeve (1863) re-described the species and illustrated a shell from the Cuming collection. The NHM collections contain a lot of two specimens from the Cuming collection with Pfeiffer’s hand written label giving the species name and collection locality. The specimen that corresponds to the shell measurements in the original description and the illustration in Reeve (1863) is figured herein (Fig. 4K).

#### 34. dautzenbergi

Taxon classificationAnimaliaArchitaenioglossaCyclophoridae

(Sykes, 1902)

Fig. 5A, B

Opisthophorus dautzenbergi Sykes, 1902a: 23. Sykes 1902b: 62, pl. 3, figs 5, 6.

##### Current generic position.

*Cyclotus* Swainson, 1840

##### Type locality.

Kelantan, Malay Peninsula [Kelantan, Malaysia].

##### Type material.

**Figure 5.**
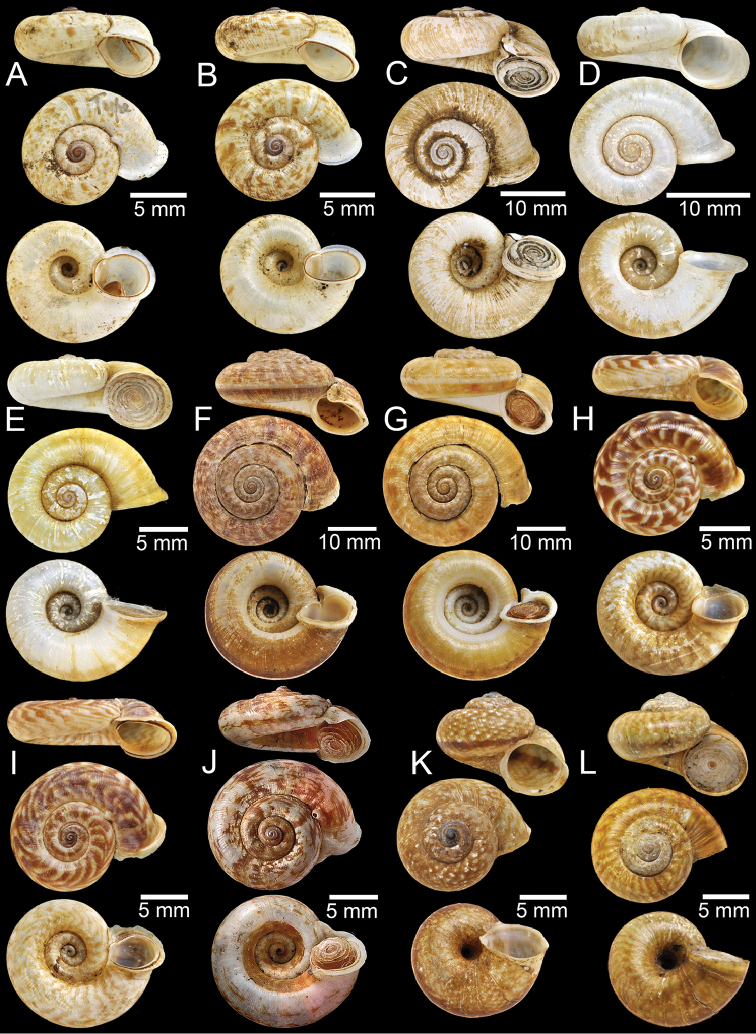
**A, B** Syntype of *Cyclotusdautzenbergi***C** paratype of *Pterocyclosdiluvium***D, E** syntype of *Cyclotusdiscoideus***F, G** syntype of *Crossopomaenganoense***H, I** syntype of *Cyclotuseuryomphalus***J** lectotype of *Pearsoniafairbanki***K, L** syntype of *Cyclotusfortunei*.

Syntype NHMUK 20170357 (3 shells; Fig. 5A, B).

##### Remarks.

The species description does not include an illustration, but later, Sykes (1902b) cited the species name and illustrated a shell of this taxon. The NHM collections contain a lot of three specimens from the Sykes collection. There is a specimen that exactly matches with the illustration in regard to the brownish zigzag streaks on the last whorl, and with the shell measurements given in the original description plus it has “Type” written on the shell. It is figured herein (Fig. 5A).

#### 35. diluvium

Taxon classificationAnimaliaArchitaenioglossaCyclophoridae

Sutcharit & Panha, 2014

Fig. 5C

Pterocyclos diluvium Sutcharit & Panha, 2014: 336, fig. 3l–p.

##### Current generic position.

*Pterocyclos* Benson, 1832

##### Type locality.

Tam Sumano, Patthalung, Thailand (7°35'183"N, 99°52'80"E).

##### Type material.

Holotype CUMZ 4595. Paratypes CUMZ 4588 (11 specimens in ethanol), NHMUK 20150078 (2 shells, Fig. 5C).

#### 36. discoideus

Taxon classificationAnimaliaArchitaenioglossaCyclophoridae

(Sowerby I, 1843)

Fig. 5D, E

Cyclostoma discoideum Sowerby I, 1843a: 111, pl. 25, figs 87, 88. Pfeiffer 1849: 144, 145, pl. 20, figs 1–3.
Cyclotus discoideus — Reeve 1863: volume 14, Cyclotus, pl. 5, species 23. Kobelt 1902: 190, 191.

##### Current generic position.

*Cyclotus* Swainson, 1840

##### Type locality.

Demerara.

##### Type material.

Syntype NHMUK 20170358 (2 shells; Fig. 5D, E).

##### Remarks.

The original description and illustration in Sowerby I (1843a) as well as those in Pfeiffer (1849) and Reeve (1863) are particularly accurate, both showing the varices on the last whorl, which suggests that these figures are from the same specimen. The NHM collections contain a lot of two shells from the Cuming collection with original labels stating the taxon name and collection locality. The specimen that corresponds to the illustrations in Sowerby I (1843a), Pfeiffer (1849) and Reeve (1863) is figured herein (Fig. 5D).

#### 37. enganoense

Taxon classificationAnimaliaArchitaenioglossaCyclophoridae

Henderson, 1898

Fig. 5F, G

Crossopoma enganoense Henderson, 1898: 17, pl. 2, figs 1–3.

##### Current generic position.

*Crossopoma* Martens, 1891

##### Type locality.

Engano [Engano Island, north Bengkulu Regency, Bengkulu Province, Indonesia].

##### Type material.

Syntype NHMUK 1898.12.5.40–41 (2 shells; Fig. 5F, G).

##### Remarks.

The original description included an illustration and a set of shell measurements. The species description was based on more than one specimen. There are two shells in the NHM type collections with an original label stating “Type lot”, taxon name, and collection locality. The specimen that matches well with the illustration and the shell dimensions given in the original description is figured herein (Fig. 5F).

#### 38. euryomphalus

Taxon classificationAnimaliaArchitaenioglossaCyclophoridae

(Pfeiffer, 1857)

Fig. 5H, I

Cyclostoma (Opisthophorus) euryomphalum Pfeiffer, 1857a [1856]: 337.
Pterocyclos euryomphalus — Reeve 1863: volume 14, Pterocyclos, pl. 5, species 29.
Cyclotus euryomphalus — Kobelt 1902: 215.

##### Current generic position.

*Cyclotus* Swainson, 1840

##### Type locality.

Borneo.

##### Type material.

Syntype NHMUK 20170351 (3 shells; Fig. 5H, I).

##### Remarks.

This species was described based on specimens from the Cuming collection, and was not illustrated in the original description. Reeve (1863) re-published the description and figured a specimen from the Cuming collection. The NHM collections contain a lot of three shells from the Cuming collection with a label in Pfeiffer’s hand written label stating the taxon name and collection locality. The specimen which closely matches the illustration in Reeve (1863) and the shell measurements given in the original description is figured herein (Fig. 5H).

#### 39. fairbanki

Taxon classificationAnimaliaArchitaenioglossaCyclophoridae

(Blanford, 1869)

Fig. 5J

Spiraculum fairbanki Blanford, 1869a: 135–137.
Pterocyclos fairbanki — Hanley and Theobald 1872: 23, pl. 49, figs 1, 2.
Pearsonia fairbanki — Kobelt 1902: 175, 176, fig. 37. Kobelt 1911: 770, 771, pl. 112, figs 12, 13. Raheem et al. 2014: 45, fig. 24f.

##### Current generic position.

*Pearsonia* Kobelt, 1902

##### Type locality.

In montibus Pulney dictis, Indiae meridionalis [Pulney Mountains, south India].

##### Type material.

Lectotype (designated by Raheem et al. 2014) NHMUK 1906.05.05.79/1 (Fig. 5J).

#### 40. feddeni

Taxon classificationAnimaliaArchitaenioglossaCyclophoridae

Blanford, 1865

Fig. 6A

Pterocyclos feddeni Blanford, 1865: 83. Hanley and Theobald 1870: 3, pl. 5, fig. 9. Hanley and Theobald 1875: 3, 53, pl. 5, fig. 9, pl. 134, fig. 1. Kobelt 1902: 166.

##### Current generic position.

*Pterocyclos* Benson, 1832

##### Type locality.

Thayet Myo, Pegu [Thayet District, Magway Division, Myanmar].

##### Type material.

**Figure 6.**
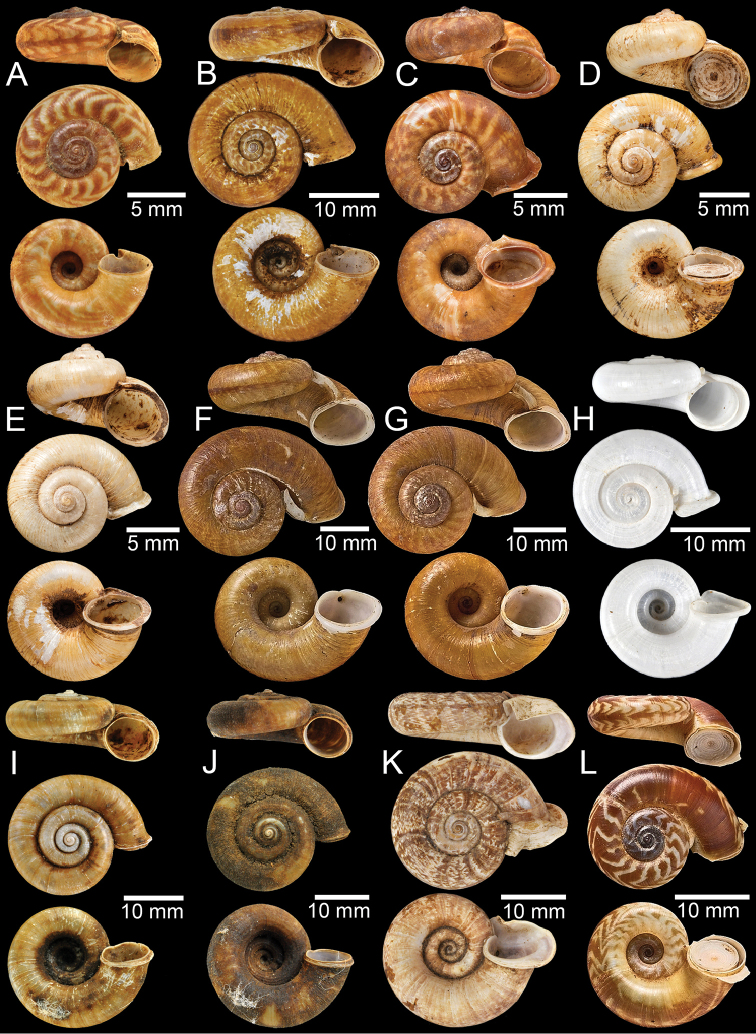
**A** Syntype of *Pterocyclosfeddeni***B** paratype of *Pterocyclosfrednaggsi***C** holotype of *Cyclotusgwendolenae***D, E** syntype of *Cyclotushainanensis***F, G** syntype of *Rhiostomahainesi***H** holotype of *Cyclotusharryleei***I, J** syntype of *Scabrinahispidula***K** holotype of *Pearsoniahuberi***L** holotype of *Cyclotushungerfordi*.

Syntype NHMUK 1906.4.4.79 (1 shell; Fig. 6A).

##### Remarks.

The original description did not clearly state how many specimens were available to the author, although only one set of measurements was given. The NHM collections contain a lot comprising one specimen and an original label stating that the specimens were figured in the Conchologia Indica. This specimen matches well with the figures in Hanley and Theobald (1875: pl. 5, fig. 9, pl. 134, fig. 1), and the measurements given in the original description.

#### 41. fortunei

Taxon classificationAnimaliaArchitaenioglossaCyclophoridae

(Pfeiffer, 1854)

Fig. 5K, L

Cyclostoma (Cyclotus) fortunei Pfeiffer, 1854b [1852]: 146.
Cyclostoma fortunei — Pfeiffer 1854a: 375, 376, pl. 49, figs 3–5.
Cyclotus foutunei — Reeve 1863: volume 14, Cyclotus, pl. 4, species 17. Kobelt 1902: 205, 206.

##### Current generic position.

*Cyclotus* Swainson, 1840

##### Type locality.

Shanghi, China [Shanghai Municipality, China].

##### Type material.

Syntype NHMUK 1980121 (1 adult + 1 juvenile; Fig. 5K, L).

##### Remarks.

This species was described from specimens in the Cuming collection and only one set of shell measurements was given in the original description. Pfeiffer (1854a) re-published the description and figured a specimen from the Cuming collection. The NHM collections contain a lot of two specimens with a label stating “Type”. The adult specimen that matches well with the illustrations in Pfeiffer (1854a) and Reeve (1863), and the shell dimensions given in the original description, is figured herein (Fig. 5K).

#### 42. frednaggsi

Taxon classificationAnimaliaArchitaenioglossaCyclophoridae

Sutcharit & Panha, 2014

Fig. 6B

Pterocyclos frednaggsi Sutcharit & Panha, 2014: 336, 337, figs 2a–c, e, 3q–s.

##### Current generic position.

*Pterocyclos* Benson, 1832

##### Type locality.

Bukit Chintamanis, Pahang, Malaysia (03°26.798'N, 102°00.987'E).

##### Type material.

Holotype CUMZ 4594. Paratypes CUMZ 4581 (18 specimens in ethanol), 4571 (29 shells), NHMUK 20150077 (2 shells, Fig. 6B).

#### 43. gwendolenae

Taxon classificationAnimaliaArchitaenioglossaCyclophoridae

(Godwin-Austen, 1889)

Fig. 6C

Rhiostoma gwendolenae Godwin-Austen, 1889: 342, pl. 36, fig. 2, 2a.
Cyclotus gwendolenae — Kobelt 1902: 215.

##### Current generic position.

*Cyclotus* Swainson, 1840

##### Type locality.

Niah Hills [Niah, Srawak, Malaysia].

##### Type material.

Holotype NHMUK 1889.12.7.9 (Fig. 6C).

##### Remarks.

Godwin-Austen clearly stated this taxon was described based on only a single specimen collected by A Everett. The original description included an illustration and a set of measurements. The NHM collections contain a Godwin-Austen type lot with an original label stating “Type”, and so we recognise this illustrated shell as the holotype fixed by monotypy.

#### 44. hainanensis

Taxon classificationAnimaliaArchitaenioglossaCyclophoridae

(Adams, 1870)

Fig. 6D, E

Pterocyclos hainanensis Adams, 1870a: 8, pl. 1, fig. 16.
Cyclotus hainanensis — Kobelt 1902: 209, 210.

##### Current generic position.

*Cyclotus* Swainson, 1840

##### Type locality.

Hainan [Hainan Province, China].

##### Type material.

Syntype NHMUK 1878.1.28.19 (3 shells; Fig. 6D, E).

##### Remarks.

Adams’s description was based on specimens collected by Mr. Swinhoe from Hainan. The original description gives both a set of shell measurements and an illustration of a specimen. The NHM collections contain a lot of three specimens from the H Adams collection with an original label stating the taxon name and collection locality. The specimen marked with an “x” on the shell matches well with the shell dimensions and illustration given in the original description and it is figured herein (Fig. 6D).

#### 45. hainesi

Taxon classificationAnimaliaArchitaenioglossaCyclophoridae

Pfeiffer, 1862

Fig. 6F, G

Rhiostoma hainesi Pfeiffer, 1862: 115, pl. 12, fig. 8. Kobelt 1902: 177. Kobelt 1911: 762, pl. 113, fig. 1.
Pterocyclos hainesi — Reeve 1863: volume 14, Pterocyclos, pl. 4, species 19.

##### Current generic position.

*Rhiostoma* Benson, 1860

##### Type locality.

Camboja [Cambodia].

##### Type material.

Syntype NHMUK 20170371 (2 shells; Fig. 6F, G).

##### Remarks.

This species was described based on material in the Cuming collection, and Pfeiffer provided an illustration and a set of measurements. The NHM type lot contains two shells collected by H Mouhot and is from the Cuming collection. It has an original label in Pfeiffer’s handwriting giving the taxon name, the collector as “Mr. Mouhot” and collection locality. The specimen that most closely matches the illustration and shell measurements given in Pfeiffers (1862) description is figured herein (Fig. 6F).

#### 46. harryleei

Taxon classificationAnimaliaArchitaenioglossaCyclophoridae

(Thach & Huber, 2018)

Fig. 6H

Spiraculum harryleei Thach & Huber, 2018: 18, 19, figs 89–92.

##### Current generic position.

*Cyclotus* Swainson, 1840

##### Type locality.

Krabi, south Thailand [Krabi Province, Thailand].

##### Type material.

Holotype NHMUK 20180248 (Fig. 6H).

##### Remarks.

The shell characters of an expanded apertural lip and a small accessory breathing device (sutural tube) located in the suture closest to the aperture suggest that it is a member of the genus *Cyclotus*. It is very closely resembles the widespread species *Cyclotussetosus* (Möllendorff, 1894) in the southern Thailand.

#### 47. hispidula

Taxon classificationAnimaliaArchitaenioglossaCyclophoridae

(Blanford, 1863)

Fig. 6I, J

Cyclophorus hispidulus Blanford, 1863: 321, 322. Hanley and Theobald 1872: 22, pl. 47, figs 5, 6.
Scabrina hispidula — Kobelt 1902: 88, fig. 22.

##### Current generic position.

*Scabrina* Blanford, 1863

##### Type locality.

Mya Leit Doung, near Ava [Myaleit Mountains, Pyinoolwin and Kyaukse Townships, Pyinoolwin and Kyaukse Districts, Mandalay Region, Myanmar].

##### Type material.

Syntype NHMUK 1906.4.4.88 (5 adults + 2 juveniles; Fig. 6I, J).

##### Remarks.

The original description did not include an illustration, and only one set of shell measurements was given. Subsequently, Hanley and Theobald (1872) figured a specimen of this species. There is a specimen in the NHM collections from the Blanford collection which was figured in Hanley and Theobald (1872) and matches well with the dimensions given in the original description, and it is figured herein (Fig. 6I).

#### 48. huberi

Taxon classificationAnimaliaArchitaenioglossaCyclophoridae

Thach, 2016

Fig. 6K

Pearsonia huberi Thach, 2016: 36, figs 48, 115–118.

##### Current generic position.

*Pearsonia* Kobelt, 1902

##### Type locality.

Thanh area, Dien Khanh District, Khanh Hao Province, Vietnam.

##### Type material.

Holotype NHMUK 20160302 (Fig. 6K).

#### 49. hungerfordi

Taxon classificationAnimaliaArchitaenioglossaCyclophoridae

(Godwin-Austen, 1889)

Fig. 6L

Rhiostoma hungerfordi Godwin-Austen, 1889: 342, 343.
Pearsonia hungerfordi — Kobelt 1902: 215.

##### Current generic position.

*Cyclotus* Swainson, 1840

##### Type locality.

Molu Hills, Borneo [Gunung Mulu, Sarawak, Malaysia].

##### Type material.

Holotype NHMUK 1891.3.17.33 (Fig. 6L).

##### Remarks.

Godwin-Austen clearly stated that this taxon was described based on only one specimen from the R Hungerford collection. There is one specimen in the NHM collections that has an original label stating “Type”. We recognise this specimen as the holotype fixed by monotypy.

#### 50. inglisianus

Taxon classificationAnimaliaArchitaenioglossaCyclophoridae

(Stoliczka, 1871)

Fig. 7A

Cyclophorus (Myxostoma) inglisianus Stoliczka, 1871: 148, 149, pl. 6, fig. 1.
Cyclophorus inglisianus — Hanley and Theobald 1876: 57, pl. 143, figs 8, 9.
Scabrina inglisianus — Kobelt and Möllendorff 1897: 88.

##### Current generic position.

*Scabrina* Blanford, 1863

##### Type locality.

Damotha, prope Moulmein [Dhammatat Cave, Mawlamyine Township, Mawlamyine District, Mon State, Myanmar].

##### Type material.

**Figure 7.**
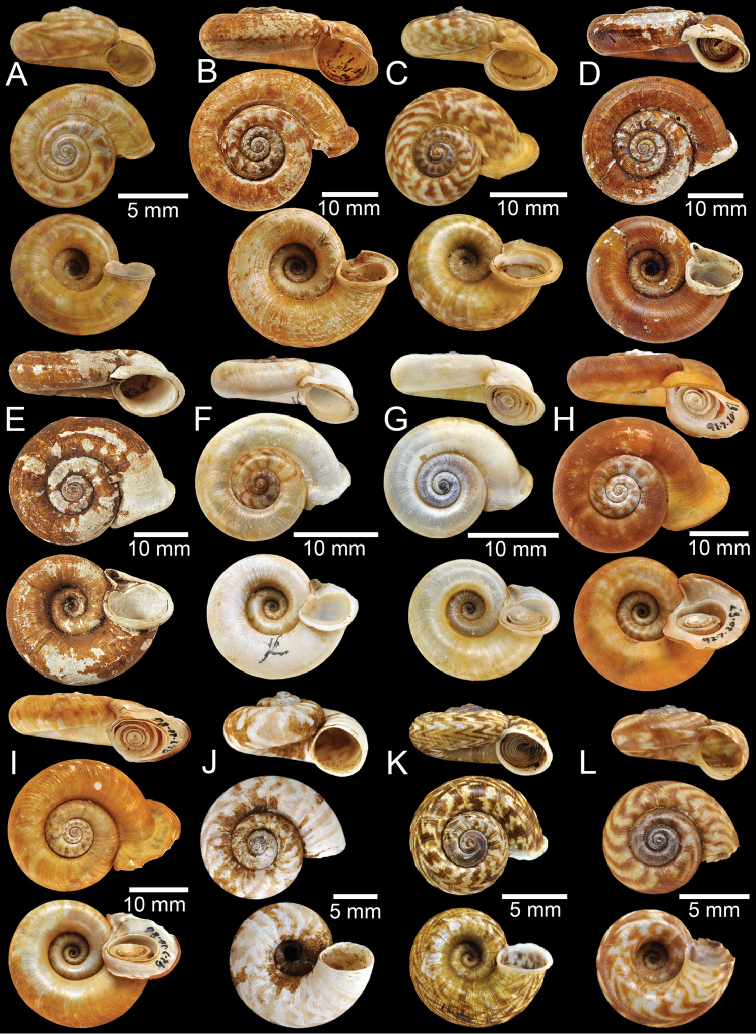
**A** Possible syntype of *Scabrinainglisianus***B** lectotype of *Pterocyclosinsignis***C** syntype of *Cyclotusiris***D, E** syntype of *Pearsoniakempi***F, G** syntype of *Cyclotuslabuanensis***H, I** syntype of *Pterocycloslatilabrum***J** paratype of *Cyclotuslepidotus***K, L** syntype of *Cyclotuslindstedti*.

Possible syntype NHMUK 20170363 (1 shell; Fig. 7A).

##### Remarks.

The species description included an illustration and one set of shell measurements. There is a shell in the NHM collections from the Blanford collection, with an original label stating “figd. Conch. Ind. 143, f. 8, 9” and the collection locality “Damatha, Molmein”. This specimen matches with the shell dimensions given in the original description but lacks an operculum. Therefore, we consider this lot to be a possible syntype.

#### 51. insignis

Taxon classificationAnimaliaArchitaenioglossaCyclophoridae

Theobald, 1865

Fig. 7B

Pterocyclos insignis Theobald, 1865: 278. Hanley and Theobald 1870: 3, pl. 5, figs 6, 7. Kobelt 1902: 166.

##### Current generic position.

*Pterocyclos* Benson, 1832

##### Type locality.

Shan State [Myanmar].

##### Type material.

Lectotype (design. n.) NHMUK 1888.12.4.1967 (Fig. 7B).

##### Remarks.

The species description was very brief and only one set of measurements was given. The NHM collections contain a mixed-species lot consisting of two specimens with an original label stating “Type”. The specimen that matches with the figures in Hanley and Theobald (1870: pl. 5, figs 6, 7), the measurements (width 29.6 mm) given in the original description and marked with an “x” is here designated as the lectotype to stabilise the name.

The other shell, NHMUK 1888.12.4.1977, differs from the lectotype in having a smaller shell width (23.6 mm), shallow suture without a canal-like structure and an expanded apertural lip without a canal-like accessory respiratory structure. We considered this shell as a distinct species, and so it excludes it from the type series.

#### 52. iris

Taxon classificationAnimaliaArchitaenioglossaCyclophoridae

(Godwin-Austen, 1889)

Fig. 7C

Rhiostoma iris Godwin-Austen, 1889: 343.
Cyclotus iris — Kobelt 1902: 215.

##### Current generic position.

*Cyclotus* Swainson, 1840

##### Type locality.

Borneo.

##### Type material.

Syntype NHMUK 1891.3.17.34 (1 shell; Fig. 7C).

##### Remarks.

The original description gave the measurements for only one shell and did not include an illustration. The NHM collections contain a lot of one specimen from the R Hungerford collection with an original label stating “Type” and giving the collection locality as “Borneo”. This specimen matches well with the original description and shell dimensions given.

#### 53. kempi

Taxon classificationAnimaliaArchitaenioglossaCyclophoridae

(Godwin-Austen, 1915)

Fig. 7D, E

Spiraculum kempi Godwin-Austen, 1915: 496, 497, pl. 39, figs 4, 4a, 5, 5a.
Pearsonia kempi — Gude 1921: 119.

##### Current generic position.

*Pearsonia* Kobelt, 1902

##### Type locality.

Abor Hills and Ponging [in the area of Arunachal Pradesh State, India].

##### Type material.

Syntype NHMUK 1903.7.1.3105 from Abor Hill (2 shells; Fig. 7D), NHMUK 1903.7.1.3047 from Ponging (2 shells; Fig. 7E).

##### Remarks.

Godwin-Austen’s description clearly indicated that the type series was from two collection localities “Abor Hills” and “Pongping”, and he provided illustrations of two shells from each different specimen lot. These two specimen lots were listed as being housed in the NHM collections. These two type lots have an original label in Godwin-Austen’s handwriting stating species name, collection locality and giving his own catalogue numbers. The figured specimen (Godwin-Austen 1915: figs 4, 4a) labelled as “Type”, is figured herein (Fig. 7D). Another figured shell (Godwin-Austen 1915: figs 5, 5a) labelled as “Typic.” is also figured herein (Fig. 7E). In addition, the original description states ‘Two specimens to Indian Museum’.

#### 54. labuanensis

Taxon classificationAnimaliaArchitaenioglossaCyclophoridae

(Pfeiffer, 1864)

Fig. 7F, G

Pterocyclos labuanensis Pfeiffer, 1864 [1863]: 525. Pfeiffer 1869: 443, pl. 98, figs 8–10.
Cyclotus labuanensis — Kobelt 1902: 212, 213, fig. 44.

##### Current generic position.

*Cyclotus* Swainson, 1840

##### Type locality.

In insula Labuan [Federal Territory of Labuan, Malaysia].

##### Type material.

Syntype NHMUK 20170369 (3 shells; Fig. 7F, G).

##### Remarks.

The species was described based on material from the Cuming collection. The original description did not include an illustration and only one set of shell dimensions was given. Pfeiffer (1869) re-published the description and illustrated a specimen. The NHM collections contain a lot of three shells from the Cuming collection with a label stated “Type”. The specimen that closely matches with the shell measurements, the illustration in Pfeiffer (1869) and has an “X” written on the shell, is figured herein (Fig. 7F).

#### 55. latilabrum

Taxon classificationAnimaliaArchitaenioglossaCyclophoridae

Smith, 1895

Fig. 7H, I

Pterocyclos latilabrum Smith, 1895: 116, pl. 3, figs 23–25. Kobelt 1902: 166.

##### Current generic position.

*Pterocyclos* Benson, 1832

##### Type locality.

Gomanton Hill, N. Borneo [Gomantong Hill, Sandakan Division, Sabah, Malaysia].

##### Type material.

Syntype NHMUK 1892.7.20.85–87 (3 shells; Fig. 7H, I).

##### Remarks.

Smith based this taxon on more than one specimen since the author states “most of the specimens”. The original description includes an illustration but no shell measurements are given. The type lot in the NHM collections contain three specimens with an original label in Smith’s handwriting. The specimen that most closely matches the illustrations in the original description is figured herein (Fig. 7H).

#### 56. lepidotus

Taxon classificationAnimaliaArchitaenioglossaCyclophoridae

Vermeulen, 1996

Fig. 7J

Cyclotus lepidotus Vermeulen, 1996: 151, fig. 3. Vermeulen and Whitten 1998: 42, fig. 18a, b.

##### Current generic position.

*Cyclotus* Swainson, 1840

##### Type locality.

Nusa Penida [Nusa Penida Island, Klungkung Regency, Bali Province, Indonesia].

##### Type material.

Holotype RMNH.MOL 57140, paratypes RMNH.MOL58921 (25 shells), NHMUK 20000249 (1 shell; Fig. 7J)

#### 57. lindstedti

Taxon classificationAnimaliaArchitaenioglossaCyclophoridae

(Pfeiffer, 1857)

Fig. 7K, L

Cyclostoma (Cyclotus) lindstedti Pfeiffer, 1857b [1856]: 391.
Cyclotus lindstedti — Reeve 1863: volume 14, Cyclotus, pl. 8, species 45. Kobelt 1902: 206.

##### Current generic position.

*Cyclotus* Swainson, 1840

##### Type locality.

Mount Ophir, Malacca [Gunung Ledang National Park, Tangkak District, Johor, Malaysia].

##### Type material.

Syntype NHMUK 20170359 (1 adult + 2 juveniles; Fig. 7K, L).

##### Remarks.

The species description did not include an illustration, and only one set of shell measurements were given. Reeve (1863) re-published the description and illustrated an adult specimen from the Cuming collection. The NHM collections contain a lot of three shells collected by FW Lindstedt from the Cuming collection with a label in Pfeiffer’s handwritten stating the taxon name and collection locality. The adult specimen matches well with the shell dimensions given in the original description, the illustration in Reeve (1863), and is figured herein (Fig. 7K).

#### 58. lombockensis

Taxon classificationAnimaliaArchitaenioglossaCyclophoridae

(Smith, 1898)

Fig. 8A

Cyclotus (Pseudocyclophorus) lombockensis Smith, 1898a: 31, pl. 2, fig. 18. Kobelt 1902: 192.

##### Current generic position.

*Cyclotus* Swainson, 1840

##### Type locality.

Rinjani Peak, 2,500 ft [at 2500 ft., Gunung Rinjani, Lombok Island, west Nusa Tenggara Province, Indonesia].

##### Type material.

**Figure 8.**
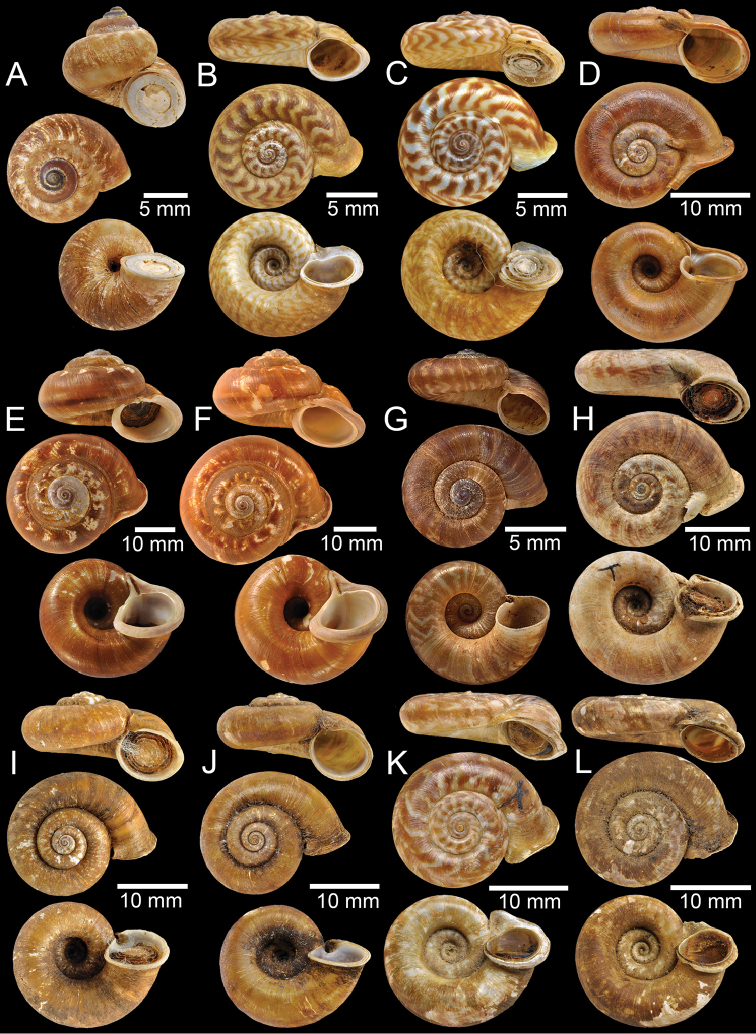
**A** Lectotype of *Cyclotuslombockensis***B, C** syntype of *Cyclotuslowianus***D** syntype of *Pearsonialuyorensis***E, F***Myxostomalychnus***E** lectotype and **F** paralectotype **G** lectotype of *Cyclotusmacalpinewoodsi***H** syntype of *Pterocyclosmagnus***I, J** syntype of *Pterocyclosmarionae***K, L** syntype of *Cyclotusmindaiensis*.

Lectotype (design. n.) NHMUK 1897.3.13.59 (Fig. 8A).

##### Remarks.

The species description was not explicitly based on one specimen, but only a single shell was illustrated and just one set of shell measurements was given in the original description. The NHM type lot contains one specimen with an original label in Smith’s handwriting and a label stating “Type”, subsequently changed to “Holotype”. This is not a valid holotype designation (ICZN 1999: Art. 73.1 and Recommendation 73F). This specimen matches well with the illustration and the shell measurements given in the original description, and is designated here as the lectotype to stabilise the name.

#### 59. lowianus

Taxon classificationAnimaliaArchitaenioglossaCyclophoridae

(Pfeiffer, 1864)

Fig. 8B, C

Pterocyclos lowianus Pfeiffer, 1864 [1863]: 526. Pfeiffer 1869: 443, pl. 98, figs 11–13.
Cyclotus lowianus — Kobelt 1902: 200.

##### Current generic position.

*Cyclotus* Swainson, 1840

##### Type locality.

In insula Labuan [Federal Territory of Labuan, Malaysia].

##### Type material.

Syntype NHMUK 20170350 (3 shells; Fig. 8B, C).

##### Remarks.

This species was described based on specimens collected by H Low from the Cuming collection. In the original description, only one set of shell measurements was given. In 1869, Pfeiffer re-described and illustrated a single specimen from the Cuming collection. The NHM collections contain a lot of three shells from the Cuming collection with Pfeiffer’s handwritten label stating the taxon name, collector and collection locality. The specimen that matches well with the shell dimensions in the original description and the illustrations in Pfeiffer (1869) is figured herein (Fig. 8B).

#### 60. luyorensis

Taxon classificationAnimaliaArchitaenioglossaCyclophoridae

(Godwin-Austen, 1915)

Fig. 8D

Spiraculum luyorensis Godwin-Austen, 1915: 500, pl. 40, figs 5, 5a, b.
Pearsonia luyorensis — Gude 1921: 119, 120.

##### Current generic position.

*Pearsonia* Kobelt, 1902

##### Type locality.

Luyor, Abor Hills [in the area of Arunachal Pradesh State, India].

##### Type material.

Syntype NHMUK 1903.7.1.3530 (1 shell; Fig. 8D).

##### Remarks.

Godwin-Austen’s description was not clearly based on only one specimen. The original description included an illustration and one set of measurements were given. The author stated that one lot of the type series was housed in the NHM collections. The specimen NHMUK 1903.7.1.3530 has a label in Godwin-Austen’s handwriting stating “Type” and exactly matches the measurements and illustrations given in the original description.

#### 61. lychnus

Taxon classificationAnimaliaArchitaenioglossaCyclophoridae

(Morelet, 1862)

Fig. 8E, F

Cyclostoma lychnus Morelet, 1862: 478. Breure et al. 2018: 334, figs 620, 621.
Myxostoma lychnus — Kobelt 1902: 86.

##### Current generic position.

*Myxostoma* Troschel, 1847

##### Type locality.

In insula Poulo-Condor [Con Son Island, Ba Ria–Vung Tau Province, Vietnam].

##### Type material.

Lectotype NHMUK 1893.2.4.501 (Fig. 8E), paralectotype NHMUK 1893.2.4.502–503 (2 shells; Fig. 8F).

##### Remarks.

The original description by Morelet (1862: 478) did not give an illustration of the species, although a set of shell dimensions was provided. The NHM register show that a lot of three specimens was purchased from Fulton from the A Morelet collection. The original label states the taxon name and gives the collection locality as “*C. breve* Martyn–*lychnus* Moret. I. Pulo Condor”. The words “All too small for types” was added at a later date, however, our measurements of all specimens are close to those shell dimensions given in the original description (width 36 mm, height 25 mm). The specimen that matches well with the shell dimensions given in the original description is here designated as the lectotype to stablise the name.

#### 62. macalpinewoodsi

Taxon classificationAnimaliaArchitaenioglossaCyclophoridae

Laidlaw, 1939

Fig. 8G

Rhiostoma macalpine–woodsi Laidlaw, 1939: 166, with text figure.

##### Current generic position.

*Rhiostoma* Benson, 1860

##### Type locality.

Sungei Siput, Perak [Sungai Siput, Kuala Kangsar District, Perak, Malaysia].

##### Type material.

Lectotype (design. n.) NHMUK 1939.4.13.23 (Fig. 8G).

##### Remarks.

The original description does not clearly state how many specimens were available to the author. Laidlaw’s use of the term “Type specimen in my collection” does not constitute a valid holotype designation (ICZN 1999: Art. 73.1.1, 73.2 and Recommendation 73F). The NHM collections hold a lot containing one specimen, NHMUK 1939.4.13.23, with a label in Laidlaw’s handwriting stating the taxon name, collection locality and “From type series”. It has subsequent been incorrectly labeled as a “Paratype”. To avoid the assumption of the existence of a holotype (ICZN 1999: Recommendation 73F), this specimen is here designated as the lectotype to stabilise the name.

#### 63. magnus

Taxon classificationAnimaliaArchitaenioglossaCyclophoridae

Godwin-Austen, 1876

Fig. 8H

Pterocyclos magnus Godwin-Austen, 1876: 174, pl. 7, figs 3, 3a, 3b. Kobelt 1902: 166, 167.

##### Current generic position.

*Pterocyclos* Benson, 1832

##### Type locality.

Dafla Hills, Assam [India].

##### Type material.

Syntype NHMUK 1903.7.1.1491 (1 shell; Fig. 8H).

##### Remarks.

This species was clearly described based on more than one specimen since the author stated “largest example”. The NHM collections contain a lot containing a single specimen with an original label in Godwin-Austen’s handwriting stating “Type”. This specimen matches well with the illustration and the measurements given in the original description.

#### 64. marionae

Taxon classificationAnimaliaArchitaenioglossaCyclophoridae

Preston, 1914

Fig. 8I, J

Pterocyclos marionae Preston, 1914: 22, with text figure.

##### Current generic position.

*Pterocyclos* Benson, 1832

##### Type locality.

Naga Hills [Assam, Arunachal Pradesh and Nagaland states, India].

##### Type material.

Syntype RBINS 524550 (1 shell; Fig. 8I), NHMUK 1911.10.12.20–21 (2 shells; Fig. 8J).

##### Remarks.

A unique name bearing type was not explicitly designated and the species description was not clearly based on one specimen. The original description included a single illustration and one set of shell measurements. The RBINS collections contain a lot with one specimen from the collection of P Dautzenberg with an original label stating “Type” and “fig.”. This specimen matches well with the shell measurements given in the original description and is figured herein (Fig. 8I). The NHM collections also contain another lot of two specimens with an original label stated “co-types”, and are also considered to be syntypes.

#### 65. mastersi

Taxon classificationAnimaliaArchitaenioglossaCyclophoridae

(Blanford, 1877)

Fig. 9A

Pterocyclos (Spiraculum) mastersi Blanford, MSS. Hanley and Theobald 1870: 3, pl. 5, fig. 1 [nomen nudum].
Spiraculum mastersi Blanford, 1877: 313, 314.
Pearsonia mastersi — Kobelt 1902: 174. Kobelt 1913: 769, 770, pl. 113, fig. 9.

##### Current generic position.

*Pearsonia* Kobelt, 1902

##### Type locality.

in montibus Naga dictis, ad latus meridionale province Assam, haud procul a Golaghat [Naga Hills, Golaghat District, southern of Assam State, India].

##### Type material.

**Figure 9.**
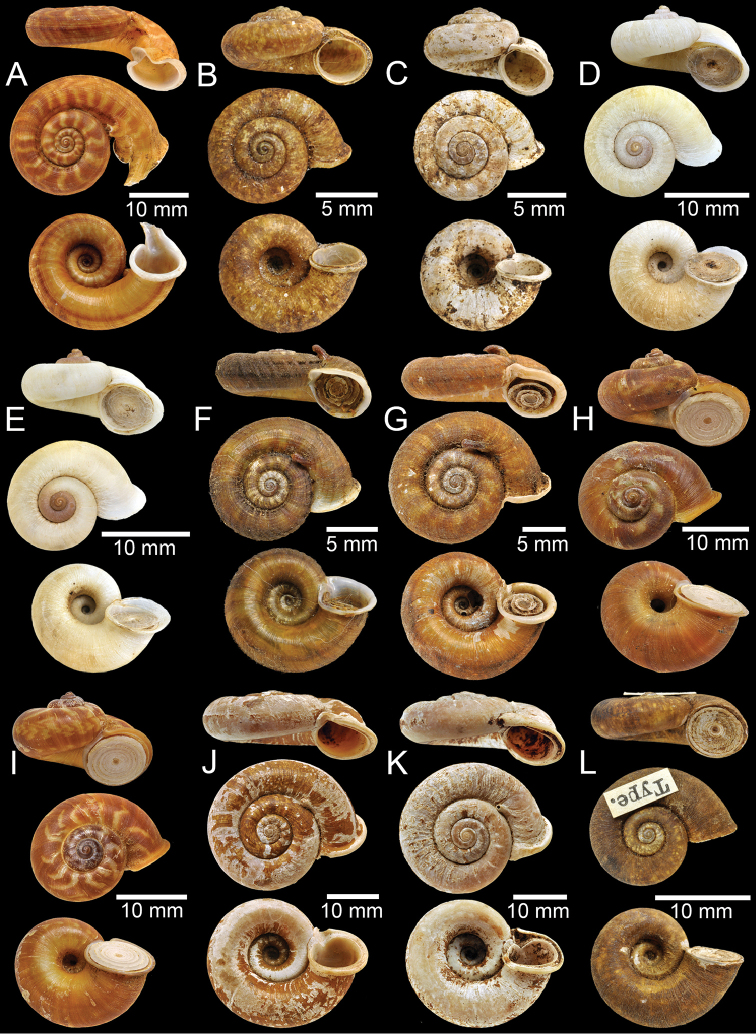
**A** Syntype of *Pearsoniamastersi***B, C***Pearsoniaminima***B** lectotype and **C** paralectotype **D, E** syntype of *Cylotusmucronatus***F, G***Pearsonianagaensis***F** lectotype and **G** paralectotype **H, I** syntype of *Cyclotusnatunensis***J, K***Pearsonianevilli***J** lectotype and **K** paralectotype **L** syntype of *Cyclotusniasensis*.

Syntype NHMUK 1888.12.4.1957 (1 shell; Fig. 9A) from Gholaghta, Assam.

##### Remarks.

This taxon was first published in Hanley and Theobald (1870), who attributed the name to WT Blanford as a manuscript name. However, Hanley and Theobald (1870: 3) did not provide any description or definition of the taxon, only a figure was provided, which does not meet the requirements of the ICZN (1999: Art. 12.2). Therefore, this taxon name was not made available. Pfeiffer (1876: 386) mentioned the taxon name without further description or definition, and so again the was not made available. Blanford (1877) published a complete description of this taxon under the same name and so making it available. Therefore, the type series is made up of the specimens sent by Blanford and cited and figured in Hanley and Theobald (1870: pl. 5, fig. 1), along with the specimens mentioned by Blanford (1870). Coan and Kabat (2012: 326) could not trace the specimen figured in Hanley and Theobald (1870: pl. 5, fig. 1). However, the NHM collections hold a lot containing a single shell from the WT Blanford collection with a handwritten label stating the taxon name, and the collection locality “Gholaghat, Assam”. This specimen matches well with the original description and the set of shell dimensions, and is considered as the syntype and figured herein.

#### 66. mindaiensis

Taxon classificationAnimaliaArchitaenioglossaCyclophoridae

(Bock, 1881)

Fig. 8K, L

Pterocyclos mindaiensis Bock, 1881: 634, pl. 55, figs 8, 8a, b.
Cyclotus mindaiensis — Kobelt 1902: 200.

##### Current generic position.

*Cyclotus* Swainson, 1840

##### Type locality.

Mindai (Amontai district) [Amuntai, Hulu Sungai Utara, South Kalimantan Province, Indonesia].

##### Type material.

Syntype NHMUK 1881.6.6.18–21 (3 adults + 1 juvenile; Fig. 8K, L).

##### Remarks.

In the original description, Bock illustrates two specimens (an adult and a juvenile), although only one set of shell measurements were given. There are four specimens in the NHM type lot from the Bock collection with an original label stating “Types”. The adult specimen, marked with an “x” matches well with the shell dimensions and illustration given in the original description (Bock 1881: fig. 8) and is figured herein (Fig. 8K).

#### 67. minima

Taxon classificationAnimaliaArchitaenioglossaCyclophoridae

(Godwin-Austen, 1915)

Fig. 9B, C

Spiraculum minimum Godwin-Austen, 1915: 501, 502, pl. 40, figs 2, 2a–c.
Pearsonia minima — Gude 1921: 121, 122.

##### Current generic position.

*Pearsonia* Kobelt, 1902

##### Type locality.

Jeku, Abor Hills and Sibbum, Abor Hills [Doje Jeku and Sibbum Villages, west Siang District, Arunachal Pradesh State, India].

##### Type material.

Lectotype (design. n.) NHMUK 1903.7.1.3145/1 (Fig. 9B) from Sibbum, Abor Hills, paralectotypes NHMUK 1903.7.1.3145/2–3 (2 shells; Fig. 9C), NHMUK 1903.7.1.3147 from Sibbum, Abor Hills (2 shells).

##### Remarks.

Godwin-Austen (1915) clearly states that this taxon was based on at least four lots of specimens from two different localities. The original description included illustrations and one set of measurements. In addition, Godwin-Austen (1915) stated that two lots were housed in the Indian Museum, and the other two lots were housed in the NHM. The specimen lot NHMUK 1903.7.1.3145 consisting of three shells has a label in Godwin-Austen’s handwriting stating “Typical”, and gives the collection locality “Sibbum”, and one of the specimens closely matches the original description. This specimen is here designated as the lectotype to stabilise the name.

The paralectotypes are the two remianing shells, NHMUK 1903.7.1.3145/2–3, from the same lot as the lectotype, the two specimens in lot NHMUK 1903.7.1.3147, and the remianing two specimen lots (nos. 6142 and 6143) housed in the Zoological Survey of India (formerly the Indian Museum) as indicated by the author. Following this lectotype designation, the type locality of this species is restricted to “Sibbum, Abor Hills” (ICZN 1999: Art. 76.2).

#### 68. mucronatus

Taxon classificationAnimaliaArchitaenioglossaCyclophoridae

(Sowerby I, 1843)

Fig. 9D, E

Cyclostoma mucronatum Sowerby I, 1843a (June): 113, pl. 25, fig. 91. Sowerby I 1843b (November): 63.
Platyrhaphe mucronata — Kobelt 1902: 185.

##### Current generic position.

*Cyclotus* Swainson, 1840

##### Type locality.

Under decayed leaves at Calauang in Luzon [Calauan, Laguna Province, Philippines].

##### Type material.

Syntype NHMUK 1842.5.10.790–798 (6 adults + 3 juveniles; Fig. 9D, E).

##### Remarks.

The species name was made available in volume 1 of the Thesaurus Conchyliorum, (Sowerby I 1843a; for date of publication see Petit (2009)). The original description includes an illustration, and the author clearly stated the taxon was described based on specimens collected by H Cuming. The NHM collections contain a lot of nine shells that have a label stating the type collection locality ‘Calauang, Luzon’. The NHM registration records show that this lot was purchased from the H Cuming collection. The specimen that closely matches with the original description and illustration in Sowerby I (1843a) is figured herein (Fig. 9D).

#### 69. nagaensis

Taxon classificationAnimaliaArchitaenioglossaCyclophoridae

(Godwin-Austen & Beddome, 1894)

Fig. 9F, G

Spiraculum nagaense Godwin-Austen & Beddome, 1894: 509.
Pearsonia nagaensis — Kobelt 1902: 174. Kobelt 1913: 771, 772, pl. 113, figs 12, 13.

##### Current generic position.

*Pearsonia* Kobelt, 1902

##### Type locality.

Maokokchung, Naga Hills [Mokokchung District, Nagaland State, India].

##### Type material.

Lectotype (design. n.) NHMUK 1903.7.1.2783 (Fig. 9F), paralectotypes NHMUK 1912.4.16.644 (3 shells; Fig. 9G).

##### Remarks.

The original description did not contain any illustrations, and only one set of measurements was given. Godwin-Austen stated that the type series was from the Beddome collection. The NHM collections contain two lots that are considered to constitute the type series. Lot NHMUK 1903.7.1.2783 consists of a single specimen, collected by Muspratt, from the Godwin-Austen collection and has original labels giving the species name “*nagaensis*”, type collection locality and stating “TYPE”. This specimen is here designated as the lectotype to stabilise the name.

The paralectotypes consist of a lot of three shells, NHMUK 1912.4.16.644, from the Beddome collection, collected by Muspratt, with an original label stating the species name “*nagaense*”, type collection locality, the reference of the original description and is marked as “PARATYPES”.

#### 70. natunensis

Taxon classificationAnimaliaArchitaenioglossaCyclophoridae

Smith, 1894

Fig. 9H, I

Cyclotus natunensis Smith, 1894a: 461, 462, pl. 16, figs 14, 14a–c. Kobelt 1902: 193.

##### Current generic position.

*Cyclotus* Swainson, 1840

##### Type locality.

Banguran [Bunguran Islands, Riau Islands Province, Indonesia].

##### Type material.

Syntype NHMUK 1894.2.1.43–44 (2 shells; Fig. 9H, I), NHMUK 1894.2.2.3–4 (2 juveniles).

##### Remarks.

The original description included an illustration and one set of shell measurements. The NHM collections contain a lot of four shells collected by A Everett from Smith’s collection with an original label stating taxon name, type collection locality, and “Type”. The specimen that matches well with the illustration and shell dimensions given in the original description is figured herein (Fig. 9H).

#### 71. nevilli

Taxon classificationAnimaliaArchitaenioglossaCyclophoridae

(Godwin-Austen, 1876)

Fig. 9J, K

Spiraculum nevilli Godwin-Austen, 1876: 174, 175, pl. 7, figs 2, 2a.
Pearsonia nevilli — Kobelt 1902: 174, 175. Kobelt 1911: 765, 766, pl. 112, figs 1, 2.

##### Current generic position.

*Pearsonia* Kobelt, 1902

##### Type locality.

near Dihiri Parbat, on the outer sandstone range [Hari Parbat, Srinagar District, Jammu and Kashmir State, India].

##### Type material.

Lectotype (design. n.) NHMUK 1903.7.1.2775/1 (Fig. 9J) and paralectotype NHMUK 1903.7.1.2775/2 (1 shell; Fig. 9K).

##### Remarks.

The species description clearly stated that the taxon was based on two specimens from Dihiri Parbat. An illustration and one set of measurements were given in the original description. The NHM type collection contains one lot, consisting of two shells with Godwin-Austen’s handwritten label stating “Type”. The specimen figured in the original description that is closest to the given measurements is here designated as the lectotype to stabilise the name.

Godwin-Austen (1915: 497, 498) also recognised two varieties as “var.” and “var. large”. Nevertheless, these two varietal names have never been made available. There are two lots of specimens labeled as “var.” and “var. large” housed in the NHM general collections, NHMUK 1903.7.1.3531 (3 shells) and NHMUK 1903.7.1.3089 (4 shells) respectively. They are excluded from the type series of this nominal species (ICZN 1999: Art. 72.4.1).

#### 72. niahensis

Taxon classificationAnimaliaArchitaenioglossaCyclophoridae

Godwin-Austen, 1889

Fig. 10B, C

Pterocyclos niahensis Godwin-Austen, 1889: 340, pl. 35, figs 3, 3a. Kobelt 1902: 167.

##### Current generic position.

*Pterocyclos* Benson, 1832

##### Type locality.

Niah Hills [Niah National Park, Miri Division, Sarawak, Malaysia].

##### Type material.

**Figure 10.**
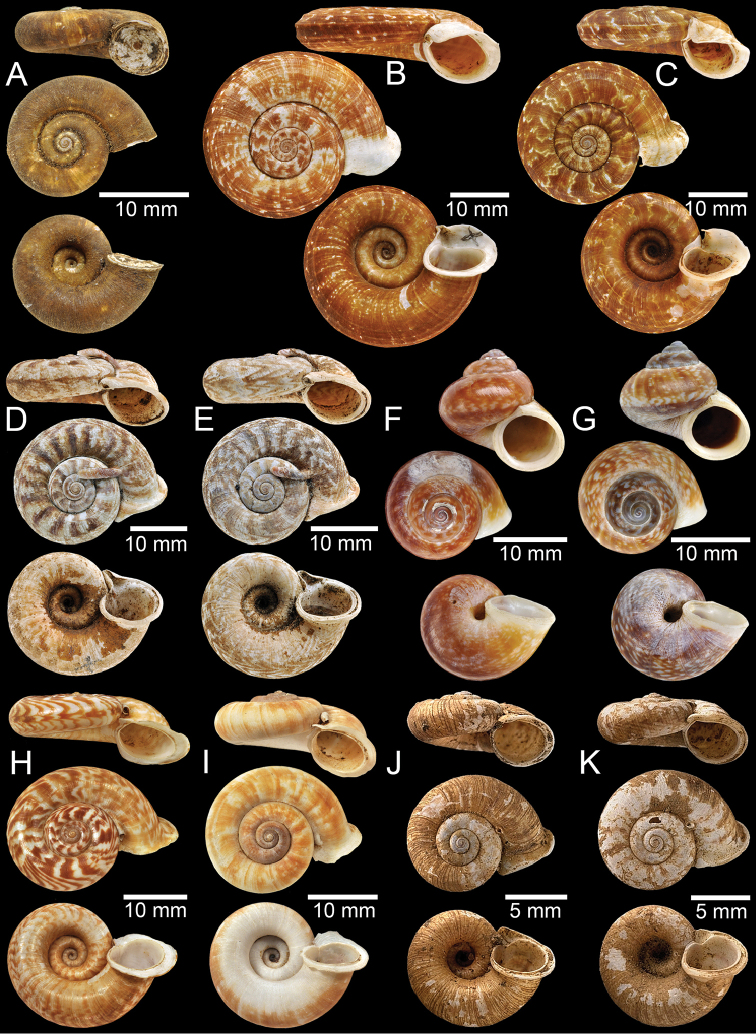
**A** Syntype of *Cyclotusniasensis***B, C** syntype of *Pterocyclosniahensis***D, E** syntype of *Pearsoniaoakesi***F, G** possible syntypes of *Cyclotuspolitus***H, I***Cyclotuspterocycloides***H** lectotype and **I** paralectotype **J, K** syntype of *Pearsoniaputaoensis*.

Syntype NHMUK 1889.12.7.16 (1 shell; Fig. 10B), NHMUK 1890.7.15.2 (1 shell; Fig. 10C).

##### Remarks.

Godwin-Austen described this species based on material from A Everett. The NHM collections contain a lot of two shells from the A Everett collection with an original label stating “Type”. The specimen which corresponds to the illustrations and shell dimensions given in the original description, and marked with an “X”, is figured herein (Fig. 10B).

Godwin-Austen also described a varietal form “var. depressa” from “Molu Hills” based on specimens collected by Mr. Boxall, ex. R Hungerford collection. The type series of the taxa could not be traced in the NHM collections.

#### 73. niasensis

Taxon classificationAnimaliaArchitaenioglossaCyclophoridae

Fulton, 1907

Figs 9L , 10A

Cyclotus niasensis Fulton, 1907: 156, pl. 10, fig. 9.

##### Current generic position.

*Cyclotus* Swainson, 1840

##### Type locality.

Nias Island, Sumatra [Nias Island, north Sumatra Province, Indonesia].

##### Type material.

Syntype NHMUK 1907.5.3.131–133 (3 shells; Figs 9L, 10A).

##### Remarks.

The original description included an illustration and gave one set of shell measurements. There are three specimens in the type lot with an original label in Fulton’s handwriting stating “Type”. The specimen that has a small label stating “Type.” glued on top of the shell matches well with the measurements and figures given in the original description, is figured herein (Fig. 9L).

#### 74. oakesi

Taxon classificationAnimaliaArchitaenioglossaCyclophoridae

(Godwin-Austen, 1915)

Fig. 10D, E

Spiraculum oakesi Godwin-Austen, 1915: 496, pl. 39, figs 3, 3a.
Pearsonia oakesi — Gude 1921: 124.

##### Current generic position.

*Pearsonia* Kobelt, 1902

##### Type locality.

Abor Hills [region in Arunachal Pradesh State, India].

##### Type material.

Syntype NHMUK 1903.7.1.3081 (3 adults + 2 juveniles; Fig. 10D, E).

##### Remarks.

The author indicated that five specimens were examined, and the original description included an illustration and one set of measurements. The NHM type collections contain a lot of five specimens with a label in Godwin-Austen’s handwritingstating “Type”. The specimen with red wool inside the aperture, illustrated in the original description and very close to the given measurements, is figured herein (Fig. 10D). The other two specimens housed in the Zoological Survey of India (no. 3081) are also considered as syntypes.

#### 75. politus

Taxon classificationAnimaliaArchitaenioglossaCyclophoridae

(Sowerby I, 1843)

Fig. 10F, G

Cyclostoma politum Sowerby I, 1843a: 97, pl. 23, fig. 17. Pfeiffer 1849: 155, pl. 21, figs 13, 14. Reeve 1862: volume 13, Cyclostoma, pl. 19, species 125.
Cyclotus politus — Kobelt 1902: 194.

##### Current generic position.

*Cyclotus* Swainson, 1840

##### Type locality.

Unknown.

##### Type material.

Possible syntype NHMUK 20170360 (4 shells; Fig. 10F, G).

##### Remarks.

The original description was published by Sowerby I in 1843 and included and illustation. Later, Pfeiffer (1849) and Reeve (1862) re-published the description and figured this species based on material from the Cuming collection. The NHM collections contain a lot of four shells from the Cuming collection with original labels giving only the taxon name. However, the collection locality “Flores (Martens in litt.)” has been subsequently added. The specimens in the Cuming collection matches well with the illustration in the original description, especially in regard to the dark brown reticulated pattern and dark spiral band on the periphery of last whorl. However, Sowerby I (1843a) did not explicitly state that the species description was based on specimens from the Cuming collection. Therefore, we consider this lot to be possible syntypes.

#### 76. pterocycloides

Taxon classificationAnimaliaArchitaenioglossaCyclophoridae

(Pfeiffer, 1855)

Fig. 10H, I

Cyclostoma (Cyclotus) pterocycloides Pfeiffer, 1855a [1854]: 300.
Pterocyclos anomalus Reeve, 1863: volume 14, Pterocyclos, pl. 5, species 27. Type locality: Borneo.
Cyclotus pterocycloides — Kobelt 1902: 216.

##### Current generic position.

*Cyclotus* Swainson, 1840

##### Type locality.

Unknown.

##### Type material.

Lectotype (design. n.) NHMUK 20170361/1 (Fig. 10H), paralectotypes NHMUK 20170361/2–3 (2 shells; Fig. 10I).

##### Remarks.

Reeve (1863) erroneously replaced the previously available name “*pterocycloides* Pfeiffer, 1855” with “*anomalus* Reeve, 1863”. This unnecessary substitution is therefore made available with it own authorship and date and is considered to be a junior objective synonym (ICZN 1999: Arts. 19.1, 33.2, 50.5 and 60.1).

Pfeiffer (1855a) stated that this species was described based on specimens from the Cuming collection. The original description did not include an illustration or collection locality, and only one set of shell measurements were given. The type lotcontains four specimens of the same species, however an original label records that this is a mixed lot containing three specimens from the Cuming collection and 1 specimen with an operculum presented by GB Sowerby I. Specimen NHMUK 20170361/1 has no operculum, is without collection locality but has an original label in Pfeiffer’s handwriting stating “*Cyclost. pterocycloides* Pfr.”. This specimen, that matches with the description and shell measurements given in the original description, and the illustration in Reeve (1863), is here designated as the lectotype to stabilise the name. The other two shells, NHMUK 20170361/2–3, from the Cuming collection lot therefore become the paralectotypes.

The remaining a single shell with an operculum, NHMUK 1886.9.10.1, which was presented by GB Sowerby I and has the collection locality “Borneo”is excluded from the type series.

#### 77. puriensis

Taxon classificationAnimaliaArchitaenioglossaCyclophoridae

Nevill, 1878

Fig. 11A

Pterocyclos rupestris var. puriensis Nevill, 1878: 260. Kobelt 1902: 169.

##### Current generic position.

*Pterocyclos* Benson, 1832

##### Type locality.

Pooree (=Puri) [Puri District, Odisha State, India] and Chandbally [region in Odisha State, India].

##### Type material.

**Figure 11.**
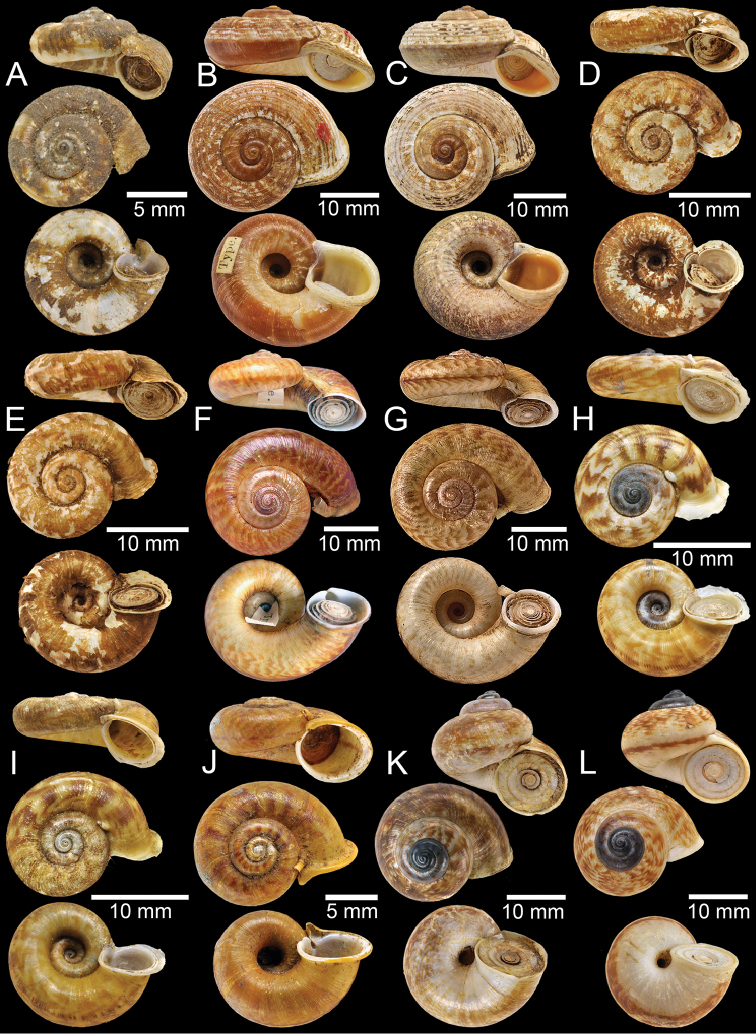
**A** Possible syntype *Pterocyclosrupestrispuriensis***B, C** syntype of *Cyclotuspyrostoma***D, E** syntype of *Cyclotussiamensis***F, G** syntype of *Rhiostomasimplicilabre***H, I** syntype of *Cyclotusspiniferus***J** holotype of *Pterocyclosspiramentum***K, L** syntype of *Cyclotussuluanus*.

Possible syntype NHMUK 1912.4.16.658 (1 shell; Fig. 11A) from Puri, Orissa.

##### Remarks.

The author clearly indicated that this taxon was described based on material from Pooree (=Puri) and Chandbally. The original description does not include any illustrations or shell measurements, and the species description is very brief, simply indicating that this taxon is a “dwarf form with raised spire…”. The NHM collections contain a lot containing one shell from the Beddome collection with an original label stating collection locality as “Puri, Orissa”. Since this lot was registered as “Nev. Hnd. List. p. 260”, we consider this shell to be a possible syntype.

#### 78. putaoensis

Taxon classificationAnimaliaArchitaenioglossaCyclophoridae

(Godwin-Austen, 1915)

Fig. 10J, K

Spiraculum putaoensis Godwin-Austen, 1915: 500, 501, pl. 40, figs 3, 3a, b.
Pearsonia putaoensis — Gude 1921: 125.

##### Current generic position.

*Pearsonia* Kobelt, 1902

##### Type locality.

Putao, Upper Burma [Putao District, Kachin State, Myanmar].

##### Type material.

Syntype NHMUK 1903.7.1.3598 (3 shells; Fig. 10J, K).

##### Remarks.

The original description included an illustration, one set of measurements, and Godwin-Austen (1915) indicated that he examined three specimens. The NHM type collections contain a lot of three specimens with a label in Godwin-Austen’s handwriting stating “Type”. The specimen closest to the given measurements and illustrations in the original description is figured herein (Fig. 10J).

#### 79. pyrostoma

Taxon classificationAnimaliaArchitaenioglossaCyclophoridae

Smith, 1896

Fig. 11B, C

Cyclotus pyrostoma Smith, 1896b: 100, 101, pl. 7, figs 1–3. Kobelt 1902: 202.

##### Current generic position.

*Cyclotus* Swainson, 1840

##### Type locality.

South Celebes, at 2000 feet [south Sulawesi Province, Indonesia].

##### Type material.

Syntype NHMUK 1896.4.30.1 (1 shell; Fig. 11B), NHMUK 1896.5.1.1–2 (2 shells; Fig. 11C).

##### Remarks.

There are three specimens in the NHM type lot with a label in Smith’s handwriting stating the species name and collection locality. One specimen has a small label “Type” attached under the shell. This specimen matches well to the illustration and shell dimensions given in the original description and is figured herein (Fig. 11B).

#### 80. siamensis

Taxon classificationAnimaliaArchitaenioglossaCyclophoridae

(Martens, 1860)

Fig. 11D, E

Opisthoporus siamensis Martens, 1860: 10.
Cyclotus siamensis — Kobelt 1902: 211.

##### Current generic position.

*Cyclotus* Swainson, 1840

##### Type locality.

Siam [Thailand].

##### Type material.

Syntype NHMUK 1856.7.21.1 (5 adults + 3 juveniles; Fig. 11D, E).

##### Remarks.

Martens (1860) states that this species was described based on material in the British Museum [= NHM] collected by JC Bowring from Siam. The original description did not include an illustration and only one set of shell measurements was given. The NHM collections contain a lot of eight specimens with an original label stating “typical specimens”, and with the collection locality “Siam”. The NHM registration book shows that this specimen lot was purchased from JC Bowring. The specimen that matches most closely with the shell measurements in the original description is figured herein (Fig. 11D).

#### 81. simplicilabre

Taxon classificationAnimaliaArchitaenioglossaCyclophoridae

Pfeiffer, 1862

Fig. 11F, G

Rhiostoma simplicilabre Pfeiffer, 1862: 115, pl. 12, fig. 7. Kobelt 1902: 178, 179. Kobelt 1911: 756, 757, pl. 110, figs 5–7, pl. 113, fig. 3.
Pterocyclos simplicilaris — Reeve 1863: volume 14, Pterocyclos, pl. 4, species 20.

##### Current generic position.

*Rhiostoma* Benson, 1860

##### Type locality.

Camboja [Cambodia].

##### Type material.

Syntype NHMUK 20130214 (4 shells; Fig. 11F, G).

##### Remarks.

This species was described based on specimens collected by H Mouhot from the Cuming collection. The original description included an illustration and one set of shell measurements. There are four specimens in the NHM collections from the Cuming collection with an original label in Pfeiffer’s handwriting stating the taxon name, collector and collection locality. The specimen that closely matches the illustration ans has a small label stating “Type” glued inside the umbilicus is figured herein (Fig. 10F).

#### 82. spiniferus

Taxon classificationAnimaliaArchitaenioglossaCyclophoridae

(Morelet, 1861)

Fig. 11H, I

Cyclostoma spiniferum Morelet, 1861: 177. Breure et al. 2018: 439, 440, figs 1082, 1083.
Cyclotus spiniferus — Kobelt 1902: 217.

##### Current generic position.

*Cyclotus* Swainson, 1840

##### Type locality.

probably in Borneo.

##### Type material.

Syntype NHMUK 1893.2.4.1547–1549 (3 shells; Fig. 11H, I).

##### Remarks.

The original description did not include an illustration and only one set of shell measurements was given. There are three specimens in the NHM collections purchased from A Morelet with the original label stating “type of spiniferum on left”, and with the collection locality “Borneo”. The specimen with an attached operculum, that most closely matched with the measurements given in the original description, and is marked with “X” under the shell, is figured herein (Fig. 10H).

#### 83. spiramentum

Taxon classificationAnimaliaArchitaenioglossaCyclophoridae

Godwin-Austen, 1915

Fig. 11J

Pterocyclos spiramentum Godwin-Austen, 1915: 498, 499, pl. 40, figs 4, 4a, 4b.

##### Current generic position.

*Pterocyclos* Benson, 1832

##### Type locality.

Abor Hills [region in Arunachal Pradesh State, India].

##### Type material.

Holotype NHMUK 1903.7.1.3082 (Fig. 11J).

##### Remarks.

The original description clearly stated that this taxon was described based on only one specimen. The NHM collections contain a lot consisting of a single specimen with a label in Godwin-Austen’s handwriting stating “Type”. This specimen matches well with the illustrations and measurements given in the original description, and so we consider this specimen as the holotype fixed by monotypy.

#### 84. subflammulatus

Taxon classificationAnimaliaArchitaenioglossaCyclophoridae

Pfeiffer, 1861

Fig. 12A

Cyclotus subflammulatus Pfeiffer, 1861: 28. Reeve 1863: volume 14, Cyclotus, pl. 8, species 43. Kobelt 1902: 194, 195.

##### Current generic position.

*Cyclotus* Swainson, 1840

##### Type locality.

Ise of Batchian [Bacan Islands, North Maluku Province, Indonesia].

##### Type material.

**Figure 12.**
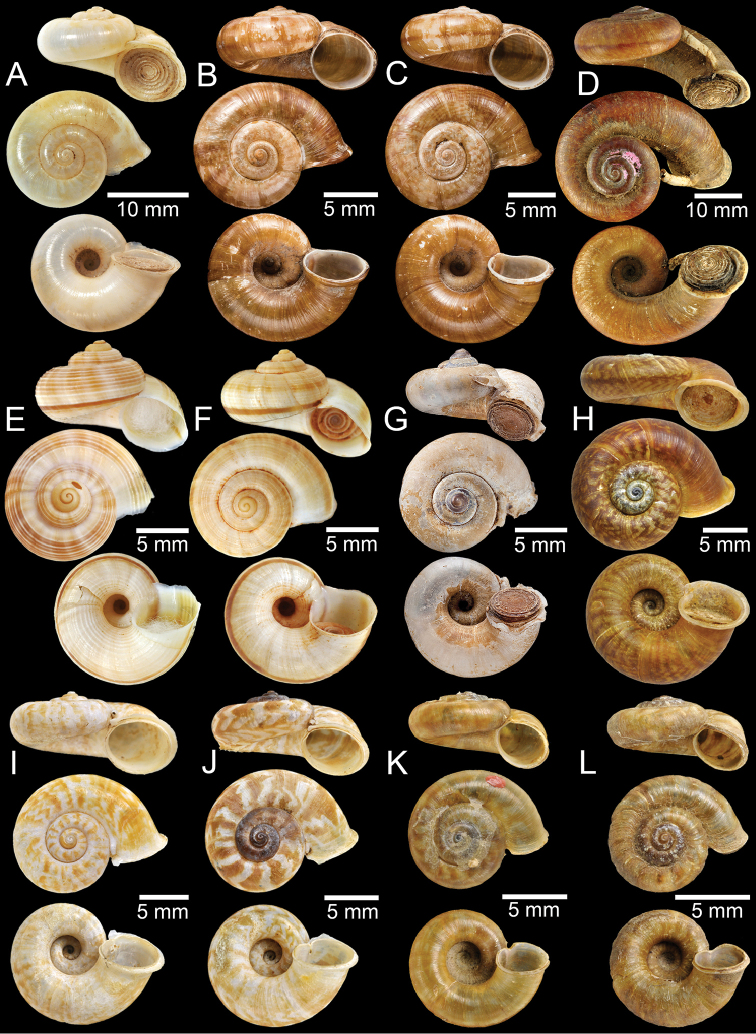
**A** Syntype of *Cyclotussubflammulatus***B, C***Cyclotustaivanus***B** lectotype and **C** paralectotype **D** paratype of *Rhiostomatomlini***E, F** syntype of *Cyclotustrailli***G** holotype of *Pearsoniatravancornica***H** syntype of *Cyclotustrusanensis***I, J** syntype of *Cyclotustubuliferus***K, L***Scabrinavanbuensis***K** lectotype and **L** paralectotype.

Syntype NHMUK 20170362 (1 shell; Fig. 12A).

##### Remarks.

The original description did not include an illustration and only one set of shell measurements was given. Pfeiffer (1861) stated that this taxon was described from a specimen collected by AR Wallace in the Cuming collection. Later Reeve (1863) re-published the description and figured a specimen from the Cuming collection. The NHM collections contain a lot consisting of only one specimen from the Cuming collection with a label in Pfeiffer’s handwriting stating the species name and collection locality, however this has been subsequently overwritten. This shell matches well with the measurements given in the original description and the illustration in Reeve (1863).

#### 85. suluanus

Taxon classificationAnimaliaArchitaenioglossaCyclophoridae

Smith, 1894

Fig. 11K, L

Cyclotus suluanus Smith, 1894b: 56, pl. 4, fig. 7. Kobelt 1902: 195.

##### Current generic position.

*Cyclotus* Swainson, 1840

##### Type locality.

Sulu [Sulu Province, Philippines].

##### Type material.

Syntype NHMUK 1891.3.17.966–967 (2 shells; Fig. 11K, L).

##### Remarks.

The species description was clearly based on two specimens collected by R Hungerford and housed in the British Museum [= NHM]. The original description included an illustration and gave one set of shell measurements. The NHM collections contain a lot of two specimens with a label stating “Types”. The specimen that matches well with the illustration and shell dimensions given in the original description is figured herein (Fig. 10K).

The manuscript name “*Cyclotus suluanus* m.” was first mentioned in Möllendorff (1890: 270) and then cited as “*Cyclotus suluanus* Möllf., MS.” in Cooke (1892). However, these two treatments do not make this name available since they lacked description or indication as is required by the ICZN (1999: Art. 12). Smith (1894) published a complete description and illustration of this taxon, where the name was made available and therefore Smith is given authorship.

#### 86. taivanus

Taxon classificationAnimaliaArchitaenioglossaCyclophoridae

Adams, 1870

Fig. 12B, C

Cyclotus taivanus Adams, 1870b: 378, 379, pl. 27, figs 11, 11a. Kobelt 1902: 207. Hwang 2014: 4, fig. 1a.

##### Current generic position.

*Cyclotus* Swainson, 1840

##### Type locality.

Taiwan, Formosa.

##### Type material.

Lectotype (designated by Hwang 2014: 4, fig. 1a) NHMUK 1871.1.20.9/1 from Formosa (Fig. 12B), paralectotypes NHMUK 1871.1.20.9/2–8 from Formosa (7 shells; Fig. 12C), NHMUK 1878.1.28.22 from Taiwan, Formosa (3 shells).

#### 87. tomlini

Taxon classificationAnimaliaArchitaenioglossaCyclophoridae

Salisbury, 1949

Fig. 12D

Rhiostoma tomlini Salisbury, 1949: 41–42, pl. 3b, figs 3, 4.

##### Current generic position.

*Rhiostoma* Benson, 1860

##### Type locality.

Khao Sabap, Siam [Plieu National Park, Khlung District, Chanthaburi Province, Thailand].

##### Type material.

Holotype NMW 1955.158.24924, paratypes NHMUK 1949.6.7.1 (2 shells; Fig. 12D), NHMUK 20170372 JE Cooper coll. Acc. no. 2150 (1 juvenile).

##### Remarks.

The original description included an illustration, one set of shell measurements, and was clearly based on more than one specimen. The author indicated that the ‘Type’ was kept in the JR le B Tomlin collection and ‘Paratypes’ were housed in the NHM collections. The holotype was clearly designated and is housed in the JR le B Tomlin collection (later transferred to the NMW in Cardiff). The NHM collections contain two lots, consisting of three shells in total, both containing an original label stating “Paratype”. The collection locality matches with the original description. Therefore, we consider these specimens as paratypes.

#### 88. trailli

Taxon classificationAnimaliaArchitaenioglossaCyclophoridae

Pfeiffer, 1862

Fig. 12E, F

Cyclotus trailli Pfeiffer, 1862: 116, fig, 4. Reeve 1863: volume 14, Cyclotus, pl. 9, species 56.

##### Current generic position.

*Cyclotus* Swainson, 1840

##### Type locality.

Russel-Canda, Madras [in the area of Chennai District, Tamil Nadu State, India].

##### Type material.

Syntype NHMUK 20030588 (3 shells; Fig. 12E, F).

##### Remarks.

The original description by Pfeiffer includes the illustration of a shell and gives one set of shell measurements. The type lot in the NHM collections was collected by “Dr. Trail” and is from the Cuming collection as stated in the original description. It has an original label in Pfeiffer’s handwriting giving the species name and collection locality. The largest specimen, marked with an “x” which most closely matches the measurements and the illustration shown in the original description is figured herein (Fig. 12E).

#### 89. travancorica

Taxon classificationAnimaliaArchitaenioglossaCyclophoridae

(Blanford, 1880)

Fig. 12G

Spiraculum travancoricum Blanford, 1880: 212, 213, pl. 3, figs 6, 6a.
Pearsonia travancornica — Kobelt 1902: 175. Raheem et al. 2014: 45, 46, fig. 25a.

##### Type locality.

In montibus Travancoricis haud procul a Tinnevelly [Hills between Kerala State and Tinnevelly District, Tamil Nadu State, India].

##### Type material.

Holotype NHMUK 1912.04.16.646/1 (Fig. 12G) fixed by monotypy.

#### 90. trusanensis

Taxon classificationAnimaliaArchitaenioglossaCyclophoridae

Godwin-Austen, 1889

Fig. 12H

Cyclotus trusanensis Godwin-Austen, 1889: 344, pl. 36, figs 5, 5a. Kobelt 1902: 203.

##### Current generic position.

*Cyclotus* Swainson, 1840

##### Type locality.

Trusan Island [Terusan Island, Sabah, Malaysia].

##### Type material.

Syntype NHMUK 1889.12.7.22 (1 shell; Fig. 12H).

##### Remarks.

The species description was clearly based on more than one specimen. The original description included an illustration of a single specimen and gave two sets of shell measurements. The NHM collections contain a lot consisting of a single shell with an original label stating “type”. This shell matches with the illustration and the shell sizes correspond well with the measurements of the specimen cited as “Size of shell drawn”, in the original description.

#### 91. tubuliferus

Taxon classificationAnimaliaArchitaenioglossaCyclophoridae

(Pfeiffer, 1854)

Fig. 12I, J

Cyclostoma (Cyclotus) tubuliferum Pfeiffer, 1854c [1853]: 51.
Pterocyclos tubuliferus — Reeve 1863: volume 14, Pterocyclos, pl. 5, species 24.
Cyclotus tubuliferus — Kobelt 1902: 218.

##### Current generic position.

*Cyclotus* Swainson, 1840

##### Type locality.

unknown.

##### Type material.

Syntype NHMUK 20170370 (3 shells; Fig. 12I, J).

##### Remarks.

The original description did not include an illustration or collection locality. Pfeiffer stated that this species was described based on material from the Cuming collection, and only one set of shell dimensions was given. Later, Reeve (1863) re-published the description and figured a specimen from the Cuming collection. A specimen lot containg three shells from the Cuming collection with an original label in Pfeiffer’s handwriting giving the species name is housed in the NHM collections. The specimen that most closely matches the measurements given in the original description and the illustration in Reeve (1863) is figured herein (Fig. 12I).

#### 92. vanbuensis

Taxon classificationAnimaliaArchitaenioglossaCyclophoridae

(Smith, 1896)

Fig. 12K, L

Pterocyclus vanbuensis Smith, 1896a: 130.
Scabrina vanbuensis — Kobelt 1902: 90, 91.

##### Current generic position.

*Scabrina* Blanford, 1863

##### Type locality.

Vanbu, Tonkin [Van Ban District, Lao Cai Province, Vietnam].

##### Type material.

Lectotype (design. n.) NHMUK 1896.1.25.7 (Fig. 12K), paralectotype NHMUK 1896.1.25.8 (1 shell; Fig. 12L).

##### Remarks.

The original description included only one set of shell measurements; however, the species description was not explicitly based on one specimen. There are two shells in the NHM type lot with an original label stating “Types”, subsequently changed to read “holotype red spot”. The shell that matches the measurements given in the original description is here designated as the lectotype to stabilise the name.

#### 93. vicinus

Taxon classificationAnimaliaArchitaenioglossaCyclophoridae

Smith, 1896

Fig. 13A, B

Cyclotus vicinus Smith, 1896c: 150, fig. 13. Kobelt 1902: 195.

##### Current generic position.

*Cyclotus* Swainson, 1840

##### Type locality.

Jampea Island [Pulau Jampea, Selayar Islands Regency, south Sulawesi Province, Indonesia].

##### Type material.

**Figure 13.**
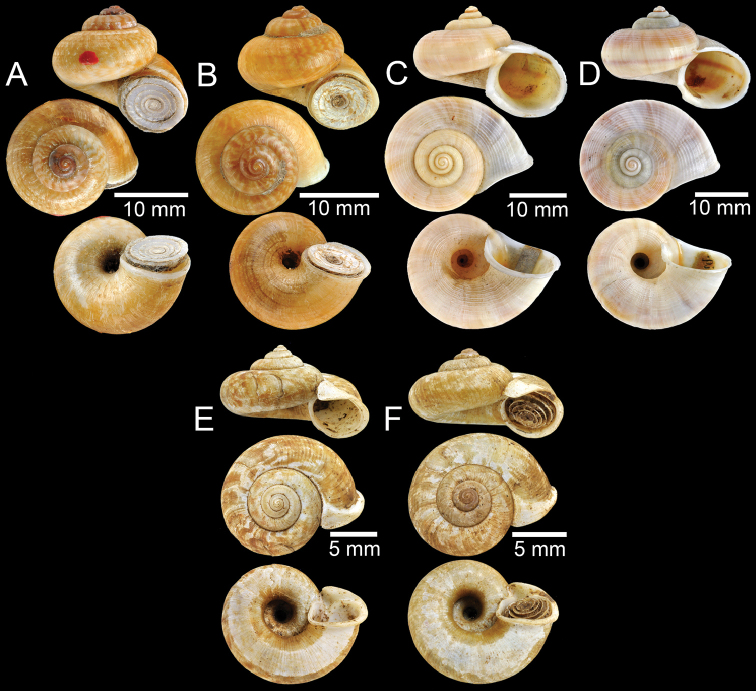
**A, B** Syntype of *Cyclotusvicinus***C, D** possible syntypes of *Cyclotusvolvuloides***E, F***Pterocyclosanamullayensis* Sutcharit and Panha, new species **E** holotype and **F** paratype.

Syntype NHMUK 1896.5.16.46–51 (5 adults + 1 juvenile; Fig. 13A, B).

##### Remarks.

Smith stated in the introduction to his paper that he had received specimens from A Everett. There are six specimens in the NHM type lot with original labels in Smith’s handwriting. The original description gives one set of measurements and an illustration of one specimen. The specimen that corresponds to the figure and measurements given in the original description, and with a red spot on the shell is figured herein (Fig. 13A).

#### 94. volvuloides

Taxon classificationAnimaliaArchitaenioglossaCyclophoridae

(Sowerby I, 1850)

Fig. 13C, D

Cyclostoma volvuloides Sowerby I, 1850: 162*, pl. 31b, figs 312, 313. Pfeiffer 1853b: 249, 250, pl. 33, figs 8, 9. Reeve 1863, volume 14, Cyclotus, pl. 4, species 19.
Cyclotus volvuloides — Kobelt 1902: 203, 204.

##### Current generic position.

*Cyclotus* Swainson, 1840

##### Type locality.

Unknown.

##### Type material.

Possible syntype NHMUK 20160354 (3 shells; Fig. 13C, D).

##### Remarks.

The original description of this species included an illustration. Later, Pfeiffer (1853b) and Reeve (1863) re-published the description and figured this species based on material in the Cuming collection. The NHM collections contain a lot of three shells from the Cuming collection with original labels giving the taxon name and citing the illustration “f. 312, 313”. The specimen in the Cuming collection matches well with the illustration in the original description, Pfeiffer (1853b) and Reeve (1863). However, Sowerby I did not clearly state that the species description was based on specimens from the Cuming collection. Therefore, we consider this lot to be possible syntypes.

### Systematics

#### Family Cyclophoridae Gray, 1847

##### Subfamily Cyclophorinae Gray, 1847

###### Tribe Pterocyclini Kobelt & Möllendorff, 1897

####### Genus *Pterocyclos* Benson, 1832

######## 95. Pterocyclos anamullayensis

Taxon classificationAnimaliaArchitaenioglossaCyclophoridae

Sutcharit & Panha sp. n.

http://zoobank.org/F963ADA8-1F4C-437E-97DB-3D41C27BD64B

Fig. 13E, F

Pterocyclos anamullayensis Beddome [unavailable name, only written on label of specimens].

######### Type material.

Collection RH Beddome, Holotype NHMUK 1912.4.16.629/1 (Fig. 13E). Paratypes NHMUK 1912.4.16.629/2–4 (3 shells; Fig. 13F) from the type locality.

######### Type locality.

Anamalais, India [Anamalai Hills (10°20'N, 76°55'E), Kerala and Tamil Nadu states, India].

######### Diagnosis.

The differences between *Pterocyclosanamullayensis* new species, *P.comatus* Beddome 1881 and *P.cyclophoroideus* Nevill, 1881 are the elevated spire, expanded lip, stout last whorl, absence of a spiral band on periphery of the last whorl, the wing-shape of upper peripheral lip near the suture and a canal-like accessory respiratory structure. In comparison, *P.comatus* and *P.cyclophoroideus* both have a depressed spire, narrow dark brown peripheral band, wide umbilicus, and slight expansion of the upper peripheral lip. The accessory respiratory structure of *P.comatus* forms a nearly closed tubular structure (see Raheem et al. 2014: fig. 23d, e), while *P.cyclophoroideus* has a nearly closed tubular to canal shaped structure (see Raheem et al. 2014: fig. 24b, c).

######### Description.

Shell small to medium, elevated conic, thickened, and widely umbilicated. Apex acute; spire elevated; suture wide and depressed; whorl 4 to 5 convex and becoming increasingly regular. Shell surface nearly smooth with thin growth lines; periostracum thin, corneous to brownish colour. Last whorl rounded and stout. Shell colour monochrome with a white or brown zigzag pattern. Aperture rounded with white lip; upper peripheral-lip widely expanded with wing shaped near suture; lower part of apertural lip thickened and absent to little expanded. Accessory respiratory structure canal-like or notch shape. Operculum calcareous, concave inside and multi-lamellae outside.

######### Etymology.

The specific epithet is derived from the type locality.

######### Distribution.

This new species known only from the type locality.

######### Remarks.

This new species was described based on the historical collections of RH Beddome from “Anamalis”. The NHMUK 1912.4.16.629 lot consists of four shells labelled as “*Pterocyclos anamullayensis* Bedd.” and “Type”, but this taxon name has previously never been published.

## Supplementary Material

XML Treatment for abletti

XML Treatment for aborensis

XML Treatment for amabilis

XML Treatment for amboinensis

XML Treatment for andersoni

XML Treatment for anguliferus

XML Treatment for aspersus

XML Treatment for assamenseis

XML Treatment for avana

XML Treatment for batchianensis

XML Treatment for bathyrhaphe

XML Treatment for beddomei

XML Treatment for bhamoensis

XML Treatment for bifrons

XML Treatment for birostris

XML Treatment for bitubifera

XML Treatment for boxalli

XML Treatment for boxalli

XML Treatment for brahmakundensis

XML Treatment for brounae

XML Treatment for calyx

XML Treatment for cambodjense

XML Treatment for celebensis

XML Treatment for chinensis

XML Treatment for christae

XML Treatment for chupingense

XML Treatment for cochinchinensis

XML Treatment for comatus

XML Treatment for confluens

XML Treatment for cucullus

XML Treatment for cumingi

XML Treatment for dalyi

XML Treatment for daucinus

XML Treatment for dautzenbergi

XML Treatment for diluvium

XML Treatment for discoideus

XML Treatment for enganoense

XML Treatment for euryomphalus

XML Treatment for fairbanki

XML Treatment for feddeni

XML Treatment for fortunei

XML Treatment for frednaggsi

XML Treatment for gwendolenae

XML Treatment for hainanensis

XML Treatment for hainesi

XML Treatment for harryleei

XML Treatment for hispidula

XML Treatment for huberi

XML Treatment for hungerfordi

XML Treatment for inglisianus

XML Treatment for insignis

XML Treatment for iris

XML Treatment for kempi

XML Treatment for labuanensis

XML Treatment for latilabrum

XML Treatment for lepidotus

XML Treatment for lindstedti

XML Treatment for lombockensis

XML Treatment for lowianus

XML Treatment for luyorensis

XML Treatment for lychnus

XML Treatment for macalpinewoodsi

XML Treatment for magnus

XML Treatment for marionae

XML Treatment for mastersi

XML Treatment for mindaiensis

XML Treatment for minima

XML Treatment for mucronatus

XML Treatment for nagaensis

XML Treatment for natunensis

XML Treatment for nevilli

XML Treatment for niahensis

XML Treatment for niasensis

XML Treatment for oakesi

XML Treatment for politus

XML Treatment for pterocycloides

XML Treatment for puriensis

XML Treatment for putaoensis

XML Treatment for pyrostoma

XML Treatment for siamensis

XML Treatment for simplicilabre

XML Treatment for spiniferus

XML Treatment for spiramentum

XML Treatment for subflammulatus

XML Treatment for suluanus

XML Treatment for taivanus

XML Treatment for tomlini

XML Treatment for trailli

XML Treatment for travancorica

XML Treatment for trusanensis

XML Treatment for tubuliferus

XML Treatment for vanbuensis

XML Treatment for vicinus

XML Treatment for volvuloides

XML Treatment for Pterocyclos anamullayensis

## References

[B1] Adam H (1870a) Descriptions of a new genus, and of eighteen new species of Mollusks. Proceedings of the Zoological Society of London 38: 5–9.

[B2] Adam H (1870b) Descriptions of ten new species of land and freshwater shells collected by Robert Swinhoe, Esq., in China and Formosa. Proceedings of the Zoological Society of London 38: 377–379.

[B3] Benson WH (1832) Account of new genus of land snails, allies to the genus *Cyclostoma*, of Lamarck; with a description of species found on the outlying rock of the Rajmahal range of hills. Journal of the Asiatic Society of Bengal 1: 11–14.

[B4] Benson WH (1856) Characters of seventeen ne forms of the Cyclostomacea from the British Provinces of Burmah, collected by W. Theobald, jun., Esq. Annals and Magazine of Natural History, Series 2, 17: 225–233. 10.1080/00222935608697501

[B5] Benson WH (1860) On *Clostophis* and *Rhiostoma*, new Burmese genera of land shells. Annals and Magazine of Natural History, Series 3, 5: 95–97. 10.1080/00222936008697183

[B6] Benthem Jutting WSS van (1948) Systematic studies on the non-marine Mollusca of the Indo-Australian archipelago. Treubia 19: 539–604.

[B7] Benthem Jutting WSS van (1959) Catalogue of the non-marine Mollusca of Sumatra and of its satellite islands. Beaufortia 7: 41–191.

[B8] Blanford WT (1863) Contributions to Indian Malacology. No. IV. Descriptions of new land shells from Ava, and other parts of Burma. Journal of the Asiatic Society of Bengal 32: 319–327.

[B9] Blanford WT (1865) Contributions to Indian Malacology. No. V. Descriptions of new land shells from Arakan, Pegu, and Ava, with notes on the distribution of described species. Journal of the Asiatic Society of Bengal 34: 69–105.

[B10] Blanford WT (1866) Contributions to Indian Malacology. No. VI. Descriptions of new land shells from the Nilgiri and Anamullay Hills, and other places in the peninsula of India. Journal of the Asiatic Society of Bengal 35: 31–42.

[B11] Blanford WT (1869a) Contributions to Indian Malacology. No. X. Descriptions of new species of Cyclophoridae, of *Ennea* and *Streptaxis* from the hills Southern and South-western India. Journal of the Asiatic Society of Bengal 38: 125–143.

[B12] Blanford WT (1869b) Descriptions of ne land and freshwater molluscan species collected by DR. John Anderson in Upper Burma and Yunan. Proceedings of the Zoological Society London 37: 444–450. 10.1111/j.1469-7998.1869.tb07354.x

[B13] Blanford WT (1877) Description of *Spiraculummastersi*. Journal of the Asiatic Society of Bengal 46: 313–314.

[B14] Blanford WT (1880) Contributions to Indian Malacology. No. XII. Descriptions of new land and freshwater shells from Southern and Western India, Burmah, the Andaman Islands. Journal of the Asiatic Society of Bengal 49: 181–222.

[B15] Blanford WT (1902) On *Rhiostomadalyi* n. sp. and *Sesaramegalodon* n. sp., obtained by the late Mr. W. M. Daly in Siam. Proceedings of the Malacological Society of London 5: 34–35.

[B16] Bock C (1881) List of land and freshwater shells collected in Sumatra and Borneo, with descriptions of new species. Proceedings of the Zoological Society of London 49: 628–635. 10.1111/j.1096-3642.1881.tb01317.x

[B17] Bouchet P Rocroi J-P (2005) Classification and nomenclator of gastropod families. Malacologia 47: 1–397.

[B18] Bouchet P Rocroi J-P Hausdorf B Kaim A Kano Y Nützel A Parkhaev P Schrödl M Strong EE (2017) Revised classification, nomenclator and typification of gastropod and monoplacophoran families. Malacologia 61: 1–526. 10.4002/040.061.0201

[B19] Breure ASH Ablett JD (2011) Annotated type catalogue of the Amphibulimidae (Mollusca, Gastropoda, Orthalicoidea) in the Natural History Museum, London. ZooKeys 138: 1–52. 10.3897/zookeys.138.1847 PMC3208519 22144852

[B20] Breure ASH Audibert C Ablett JD (2018) Pierre Marie Arthur Morelet (1809–1892) and his contributions to malacology. Nederlandse Malacologische Vereniging, Leiden, 544 pp.

[B21] Bullen RA (1906) On some land and freshwater Mollusca from Sumatra. Part II. Proceedings of the Malacological Society of London 7: 126–130.

[B22] Coan EV Kabat AR (2012) The malacological works and taxa of Sylvanus Hanley (1819–1899). Malacologia 55: 285–359. 10.4002/040.055.0208

[B23] Cooke AH (1892) On the geographical distribution of the Land-Mollusca of the Philippine Islands and their relations to the Mollusca of the neighbouring groups. Proceedings of the Zoological Society of London 60: 447–469. 10.1111/j.1469-7998.1892.tb06837.x

[B24] Dance SP (1986) A history of shell collecting. EJ Brill-Dr. W. Backhuys, Leiden, 265 pp.

[B25] Dickinson EC (2005) The Proceedings of the Zoological Society of London, 1859–1900: an exploration of breaks between calendar years of publication. Journal of Zoology, London 266: 427–430. 10.1017/S0952836905007077

[B26] Duncan FM (1937) On the dates of publication of the Society’s ‘Proceedings’1859–1926, with an appendix containing the dates of publication 1830–1858, compiled by F.H. Waterhouse; also of the ‘Transactions’ 1833–1869 by Henry Peavot, originally published in PZS 1893,1913. Proceedings of the Zoological Society of London, Series A 107: 71–84.

[B27] Egorov R (2009) A review of the genera of the recent terrestrial pectinibranch molluscs (synopsis mainly based on published data). Littoriniformes: Hainesiidae, Aciculidae, Cyclophoridae, Craspedopomatidae. Treasure of Russian Shells, Supplement 3, Part 2: 1–57.

[B28] Eydoux F Souleyet LFA (1852) Voyage autour de Monde exécuté pendant les années 1836 et 1837 sur la corvette La Bonite, Zoologie: Mollusques. Arthus Bertrand, Éditeur, Paris, 1–633.

[B29] Foon JK (2016) *Myxostomapetiverianumtenggolensis* (Gastropoda: Caenogastropoda: Cyclophoridae), a new subspecies of land snail from Peninsular Malaysia. Raffles Bulletin of Zoology 64: 329–334.

[B30] Fulton H (1900) A new species of *Bulimulus* from Costarica and a new *Spiraculum* from Assam. Nautilus 14: 87–88.

[B31] Fulton H (1905) On new species of *Helicarion*, *Ariophanta*, *Eulota*, Cyclotus (Eucyclotus), *Lagochilus*, and Diplommatina (Gastroptychia) Annals and Magazine of Natural History, Series 7, 16: 91–94. 10.1080/03745480509443641

[B32] Fulton H (1907) Descriptions of *Trochomorpha*, *Cochlostyla*, *Amphidromus*, *Bulimulus*, *Drymaeus*, *Placostylus*, *Stenogyra*, *Leptopoma*, *Cyclophorus*, *Cyclotus*, and *Alycaeus* Annals and Magazine of Natural History, Series 7, 19: 149–157. 10.1080/00222930709487245

[B33] Godwin-Austen HH (1876) On the Cyclostomacea of the Dafla Hills, Assam. Journal of the Asiatic Society of Bengal 45: 171–184.

[B34] Godwin-Austen HH (1889) On a collection of land shells made in Borneo by Mr. A. Everett, with descriptions of supposed new species. Part 1. Cyclostomaceae. Proceedings of the Zoological Society of London 57: 332–355.

[B35] Godwin-Austen HH (1893) On a supposed new species of *Rhiostoma* from Borneo, and notices of two other species of shells from Palawan. Annals and Magazine of Natural History, Series 6, 12: 32, 33. 10.1080/00222939308677570

[B36] Godwin-Austen HH (1915) Zoological results of the Arbor expedition, XXXIX Mollusca III. Cyclophoridae (In part). Records of the Indian Museum, Calcutta 8: 493–503.

[B37] Godwin-Austen HH Beddome RH (1894) New species of *Cyclophorus* and *Spiraculum* from the Khasi and Naga Hills, Assam. Annals and Magazine of Natural History, Series 6, 13: 508–509. 10.1080/00222939408677742

[B38] Gray JE (1847) A list of the genera of recent Mollusca, their synonyms and types. Proceedings of the Zoological Society of London 15: 129–219.

[B39] Gray JE (1855) List of Mollusca and shells in the collection of the British Museum, collected and described by Eydoux and Souleyet. Printed by order of the Trustees, London, 27 pp.

[B40] Gude GK (1921) Mollusca III, land operculates (Cyclophoridae, Truncatellidae, Assimineidae, Helicinidae). In: Shipley AS, Marshall GAK (Eds) The Fauna of British India including Ceylon and Burma. Taylor and Francis, Red Lion Court, Fleet Street, London, 386 pp.

[B41] Hanley SCT Theobald W (1870–1876) Conchologia Indica: Illustrations of the Land and Freshwater Shells of British India. London, i–xviii + 1–65 pp., 160 pl [pp 1–18, pls 1–40 (1870); pp 19–28, pls 41–60 (1872); pp 29–34, pls 61–80 (1873); pp 35–48, pls 81–120 (1874); pp 49–56, pls 121–140 (1875); pp 57–65, pls 141–160 (1876)] [Published in parts, dates after Prashad (1927)]

[B42] Henderson JB (1898) A list of land and fresh water shells of Engano with description of new species. Nautilus 12: 13–17.

[B43] Hwang C-C (2014) Annotated type catalogue of land snails collected from Taiwan (Formosa) in the Natural History Museum, London. ZooKeys 428: 1–28. 10.3897/zookeys.428.8061 PMC4140505 25901108

[B44] ICZN (International Commission on Zoological Nomenclature) (1999) International Code of Zoological Nomenclature, 4^th^ [May 2013] International Trust for Zoological Nomenclature, London, 306 pp.

[B45] Kobelt W (1902) Cyclophoridae. Das Tierreich 16: 1–663.

[B46] Kobelt W (1911 [1911–1914]) Die Gedeckekten Lungenschnecken (Cyclostomacea). In Abbildungen nach der Natur mit Beschreibungen. Dritte Abteilung. Cyclophoridae II. Systematisches Conchylien-Cabinet von Martini und Chemnitz 1(19)[(3)]: 713–1048, pls 104–156. [pp 713–816, pls 104–121 (1911)].

[B47] Kobelt W Möllendorff OF von (1897) Katalog der gegenwärtig lebend bekannten Pneumonopomen. Nachrichtsblatt der Deutschen Malakozoologischen Gesellschaft 29: 105–120.

[B48] Laidlaw FF (1939) A new *Rhiostoma* from Malaya. Journal of Conchology 21: 166.

[B49] Lee Y-C Lue K-Y Wu W-L (2012) The phylogeny and morphological adaptations of *Cyclotustaivanus* ssp. (Gastropoda: Cyclophoridae). Malacologia 55: 91–105. 10.4002/040.055.0106

[B50] Martens E von (1860) On the Mollusca of Siam. Proceedings of the Zoological Society of London 28: 6–18 [Published in parts, dates follow Dickinson (2005)]

[B51] Martens E von (1891) Landschnecken des indischen Archipels. In: Weber M (Ed.) Zoologische Ergebnisse einer Reise nach Nlederländisch Ostindien, Band 2 [1892], 209–264.

[B52] Marzuki ME bin Clements GR (2013) A new species of cyclophorid snail (Mollusca: Prosobranchia) from Terengganu, Penensular Malaysia. Raffles Bulletin of Zoology 61: 21–24.

[B53] Möllendorff OF von (1890) Die Landschnecken-Fauna der Insel Cebu. Bericht über die Senckenbergische Naturforschende Gesellschaft in Frankfurt-am-Main 1890: 189–292.

[B54] Möllendorff OF von (1894) On a collection of land-shells from the Samui Islands, Gulf of Siam. Proceedings of the Zoological Society of London 62: 146–156.

[B55] Morelet A (1861) Diagnoses de trois Cyclostomes nouveaux. Journal de Conchyliologie 9: 176–177.

[B56] Morelet A (1862) Diagnoses testarum Indo-Sinarum. Revue et Magasin de Zoologie Pure et Appliquée, Series 2, 14: 477–481.

[B57] Morelet A (1875) Series conchyliologiques, comprenant ľenumération de mollusques, terrestres et fluviatiles recueillis pendant le cours de différents voyages, ainsi que la description de plusieurs espèces nouvelles. IV. 4^e^ livraison Indo Chine, 227–377. 10.5962/bhl.title.11458

[B58] Morelet A (1881) Malacologie des Comores. Récolte de M. Marie à ľîle Mayotte. Journal de Conchyliologie 29: 212–241.

[B59] Nantarat N Sutcharit C Tongkerd P Ablett J Naggs F Pnaha S (2014a) An annotated catalogue of type specimens of the land snail genus *Cyclophorus* Monfort, 1810 (Caenogastropoda, Cyclophoridae) in the Natural History Museum, London. ZooKeys 411: 1–56. 10.3897/zookeys.411.7258 PMC4042817 24899854

[B60] Nantarat N Tongkerd P Sutcharit C Naggs F Wade CM Panha S (2014b) Phylogenetic relationships of the operculate land snail genus *Cyclophorus* Montfort, 1810 in Thailand. Molecular Phylogenetics and Evolution 70: 99–111. 10.1016/j.ympev.2013.09.013 24076249

[B61] Nantarat N Wade CM Jeratthitikul E Sutcharit C Panha S (2014c) Molecular evidence for cryptic speciation in the *Cyclophorusfulguratus* (Pfeiffer, 1854) species complex (Caenogastropoda: Cyclophoridae) with description of new species. PLoS ONE 9: e109785. 10.1371/journal.pone.0109785 PMC4192354 25299674

[B62] Nevill G (1878) Hand List of Mollusca in the Indian Museum, Calcutta. Part 1. Gastropoda. Pulmonata and Prosobranchia-Neurobranchia. Indian Museum, Calcutta, 338 pp.

[B63] Nevill G (1881) New or little-known Mollusca of the Indo-Malayan Fauna. Journal of the Asiatic Society of Bengal 50: 125–146.

[B64] Oheimb PV von Oheimb KCM von Hirano T Do TV Luong HV Ablett J Pham SV Naggs F (2018) Competition matters: Determining the drivers of land snail community assembly among limestone karst areas in northern Vietnam. Ecology and Evolution 8: 4136–4149. 10.1002/ece3.3984 29721286 PMC5916308

[B65] Petit RE (2007) Lovell Augustus Reeve (1814–1865): Malacological author and publisher. Zootaxa 1648: 1–120. 10.11646/zootaxa.1648.1.1

[B66] Petit RE (2009) George Brettingham Sowerby, I, II & III: their conchological publications and Molluscan taxa. Zootaxa 2189: 1–218.

[B67] Pfeiffer L (1849 [1843–1850]) Die gedeckelten Lungenschnecken. (Helicinacea et Cyclostomacea.). In Abbildungen nach der Natur mit Beschreibungen. Zweite Abtheilung der gedeckelten Cölopnoen. Cyclostomaceen. Systematisches Conchylien-Cabinet von Martini und Chemnitz, Ersten Bandes, neunzehnte Abtheilung, erster Theil. Bauer & Raspe, Nürnberg, 1 (19) [(1)]: i–iv, 1–288, pls A, 1–30. [p. 97–208, pls 26–30 (1849)] [Published in parts, dates follow Welter-Schultes (1999)]

[B68] Pfeiffer L (1851) Uebersicht der Gattung *Pterocyclos* Bens. Zeitschrift für Malakozoologie 8: 1–10.

[B69] Pfeiffer L (1853a) Catalogue of Phaneropneumona or terrestrial operculated Mollusca in the collection of the British Museum. Woodfall & Kinder, London, 324 pp.

[B70] Pfeiffer L (1853b, 1854a Die gedeckelten Lungenschnecken. (Helicinacea et Cyclostomacea.). Abbildungen nach der Natur mit Beschreibungen. Zweite Abtheilung der gedeckelten Cölopnoen. Cyclostomaceen. Zweite Abtheilung. In: Systematisches Conchylien-Cabinet von Martini und Chemnitz, Ersten Bandes, neunzehnte Abtheilung, zweiter Theil. [1 (19) (2)]: 229–400, pls 31–50 [pp. 229–268, pls 31–36 (1853); pp 269–400, pls 37–50 (1854)] [Published in parts, dates follow Welter-Schultes 1999]

[B71] Pfeiffer L (1854b) Descriptions of fourteen new species of operculated land shells, from the Cuming’s collection. Proceedings of the Zoological Society of London 20 [1852]: 144–147. [Published in parts, dates follow Duncan (1937)]

[B72] Pfeiffer L (1854c) Descriptions of twenty-three new species of land shells, from the collection of H. Cuming, Esq. Proceedings of the Zoological Society of London 21 [1853]: 48–54. [Published in parts, dates follow Duncan (1937)]

[B73] Pfeiffer L (1855a) Descriptions of eighteen new species of Cyclostomacea, from Mr. Cuming’s collection. Proceedings of the Zoological Society of London 22 [1854]: 299–303. [Published in parts, dates follow Duncan (1937)]

[B74] Pfeiffer L (1855b) Descriptions of thirty-eight new species of land shells, from the collection of H. Cuming, Esq. Proceedings of the Zoological Society of London 23: 111–119.

[B75] Pfeiffer L (1857a) Descriptions of sixteen new species of Pneumonopoma, from the collection of H. Cuming, Esq. Proceedings of the Zoological Society of London 24 [1856]: 336–339. [Published in parts, dates follow Duncan (1937)]

[B76] Pfeiffer L (1857b) Descriptions of thirty-three new species of land shells, from the collection of H. Cuming, Esq. Proceedings of the Zoological Society of London 24 [1856]: 385–393. [Published in parts, dates follow Duncan (1937)]

[B77] Pfeiffer L (1858) Monographia Pneumonopomorum viventium. Supplementum primum, Volume 2. Cassel, 249 pp.

[B78] Pfeiffer L (1860a) Descriptions of thirty-six new species of land shells, from Mr. H. Cuming’s, collection. Proceedings of the Zoological Society of London 28: 133–141.

[B79] Pfeiffer L (1860b [1860–1866]) Novitates Conchologicae. Series Prima. Mollusca Extramarina. Band 2. Casssel, Verlag von Theodor Fischer, 139–303, pls 37–72. [pp 139–160, pls 37–42 (1860)] [Published in parts, dates follow Johnson (1969)]

[B80] Pfeiffer L (1861) Descriptions of forty-seven new species of land shells, from the collection of H. Cuming, Esq. Proceedings of the Zoological Society of London 29: 20–29.

[B81] Pfeiffer L (1862) Description of 8 new species of Cyclostomacea from the collection of H. Cuming. Esq. Proceedings of the Zoological Society of London 30: 115–117. 10.1111/j.1469-7998.1862.tb06469.x

[B82] Pfeiffer L (1864) Descriptions of fifteen new species of land shells, from the collection of H. Cuming, Esq. Proceedings of the Zoological Society of London 31 [1863]: 523–526. [Published in parts, dates follow Duncan (1937)]

[B83] Pfeiffer L (1865) Monographia Pneumonopomorum viventium. Supplementum secundum, Volume 3. Cassel, 284 pp 10.5962/bhl.title.10621

[B84] Pfeiffer L (1869 [1866–1869]) Novitates Conchologicae. Series prima. Mollusca extramarina. Descriptions et figures de coquilles, estramarines nouvelles, ou peu connues. Beschreibung und Abbidung neuer order kritischer Land- und Süsswasser Mollusken. Tome 3. Casssel, (Th. Fischer), 301–312, pls 73–108. [pp 431–510, pls 97–108 (1869)] [Published in parts, dates follow Johnson (1969)]

[B85] Pfeiffer L (1876) Monographia Pneumonopomorum viventium. Supplementum tertium, Volume 4. Cassel, 479 pp 10.5962/bhl.title.10624

[B86] Pholyotha A Sutcharit C Panha S (2018) The land snail genus *Macrochlamys* Gray, 1847 from Thailand, with descriptions of five new species (Pulmonata: Ariophantidae). Raffles Bulletin of Zoology 66: 736–781.

[B87] Prasankok P Tongkerd P Sutcharit C Panha S (2011) Genetic divergence in the snorkel snail, *Rhiostomahousei*, a species complex in Thailand (Caenogastropoda: Cyclophoridae). Biochemical Systematics and Ecology 39: 834–840. 10.1016/j.bse.2011.08.005

[B88] Prashad B (1927) On the dates of publication of Hanley and Theobald’s “Conchologica Indica”. Journal and Proceedings of the Asiatic Society of Bengal, new series 22: 129–130.

[B89] Preston HB (1914) New land and freshwater shells from the Naga Hills, Assam. Proccedings of the Malacological Society of London 11: 19–24.

[B90] Raheem DC Taylor H Ablett J Preece RC Aravind NA Naggs F (2014) A systematic revision of the land snails of the Western Ghats of India. Tropical Natural History, Supplement 4: 1–294.

[B91] Rees WJ (1964) A review of breathing devices in land operculated snails. Proceedings of the Malacological Society of London 36: 55–67.

[B92] Reeve LA (1861) Conchologia Iconica: Illustrations of the shells of molluscous animals. Volume 13, Monograph of the genus *Cyclophorus*, pls 1–20. Lovell Reeve & Co., Henrietta Street, Covent Garden, London. [Published in parts, dates follow Petit (2007)]

[B93] Reeve LA (1862) Conchologia Iconica: Illustrations of the shells of molluscous animals. Volume 13, Monograph of the genus *Cyclostoma*, pls 18–23 and *Leptopoma*, pls 1–8. Lovell Reeve & Co., Henrietta Street, Covent Garden, London. [Published in parts, dates follow Petit (2007)]

[B94] Reeve LA (1863) Conchologia Iconica: Illustrations of the shells of molluscous animals. Volume 14, Monograph of the genus *Cyclotus*, pls 1–9 and *Pterocyclos*, pls 1–5. Lovell Reeve & Co., Henrietta Street, Covent Garden, London. [Published in parts, dates follow Petit (2007)]

[B95] Salisbury AE (1949) A new species of *Rhiostoma*. Proceedings of the Malacological Society of London 28: 41–42.

[B96] Smith EA (1878) Descriptions of new land shells from Japan and Borneo. Proceedings of the Zoological Society of London 46: 495–499. 10.1111/j.1469-7998.1878.tb07986.x

[B97] Smith EA (1894a) On the land-shells of the Natuna Islands. Annals and Magazine of Natural History, Series 6, 13: 453–465. 10.1080/00222939408677737

[B98] Smith EA (1894b) On the land-shells of the Sulu Archipelago. Annals and Magazine of Natural History, Series 6, 13: 48–60. 10.1080/00222939408677664

[B99] Smith EA (1895) On a collection of land-shells from Sarawak, British North Borneo, Palawan, and other neighboring islands. Proceedings of the Malacological Society of London 3: 97–127.

[B100] Smith EA (1896a) Notes on some land-shells from Vanbu, Tonkin, with descriptions of two new species. Annals and Magazine of Natural History, Series 6, 17: 128–130. 10.1080/00222939608680338

[B101] Smith EA (1896b) On a collection of land-shells from Celebes. Proceedings of the Malacological Society of London 2: 94–103.

[B102] Smith EA (1896c) On a collection of land-shells from the Islands of Selayar, Jampea, and Kalao. Annals and Magazine of Natural History, Series 6, 18: 144–152. 10.1080/00222939608680425

[B103] Smith EA (1898a) A list of land-shells of the island of Lombok, with descriptions of new species. Proceedings of the Malacological Society of London 3: 26–32.

[B104] Solem A (1959) Zoogeography of the land and fresh-water Mollusca of the New Hebrides. Fieldiana Zoology 43: 243–333.

[B105] Souleyet LFA (1841) Description de deux espéces nouvelles des genres Hélice et Cyclostome. Revue Zoologique par la Société Cuvierienne 4: 347.

[B106] Sowerby I GB (1843a) Thesaurus Conchyliorum, or monographs of genera of shells, Volume 1, Part 3, *Cyclostoma*. pp. 89–156, pls 23–31. [June] [Published in parts, dates follow Petit (2009)]

[B107] Sowerby I GB (1843b) Descriptions of new species of shells belonging to the genus *Cyclostoma*. Proceedings of the Zoological Society of London 11: 59–66. [November]

[B108] Sowerby I GB (1850) Thesaurus Conchyliorum, or monographs of genera of shells, Volume 2, Part 11, Additions to *Cyclostoma*. 157*–168*, pls 31A, 31B. [Published in parts, dates follow Petit (2009)]

[B109] Stanisic J (1998) Superfamily Cyclophoridea. In: Beesley PL Roos GJB Wells A (Eds) Mollusca: The Southern Synthesis, Fauna of Australia, Volume 5, Part B.CSIRO Publishing, Melbourne, 703–706.

[B110] Stoliczka F (1871) Notes on terrestrial Mollusca from the neighbourhood of Moulmein (Tenasserim Province), with description of new species. Journal of the Asiatic Society of Bengal 40: 143–177.

[B111] Sutcharit C Tongkerd P Panha S (2014) The land snail genus *Pterocyclos* Benson, 1832 (Caenogastropoda: Cyclophoridae) from Thailand and Peninsular Malaysia, with description of two new species. Raffles Bulletin of Zoology 62: 330–338.

[B112] Sutcharit C Ablett J Tongkerd P Naggs F Panha S (2015) Illustrated type catalogue of *Amphidromus* Albers, 1850 in the Natural History Museum, London, and descriptions of two new species. ZooKeys 492: 49–105. 10.3897/zookeys.492.8641 PMC4389215 25878542

[B113] Swainson W (1840) Treatise on Malacology; or the Natural Classification of Shells and Shell-Fish. Printed for Longman, Orme, Brown, Green & Longmans, London, 419 pp.

[B114] Sykes ER (1898) List of the species of *Cataulus* found in Ceylon, with descriptions of some new land-shells from that island. Proceedings of the Malacological Society of London 3: 65–74.

[B115] Sykes ER (1902a) Descriptions of six new land shells from the Malay Peninsula. Journal of Malacology 9: 22–23.

[B116] Sykes ER (1902b) On a collection of land and fresh water shells from Kelantan, Malay Peninsula. Journal of Malacology 9: 60–63.

[B117] Thach NN (2016) Vietnamese New Mollusks: Sea Shells, Land Snails and Cephalopods. 48HrBooks Compang, USA, 205 pp.

[B118] Thach NN (2018) New shells of South Asia: Seashells-Landsnails-Freshwater shells, 3 new genera, 132 new species & subspecies. 48HrBooks Company, USA, 173 pp.

[B119] Theobald W (1865) Notes on a collection of land and freshwater shells from the Shan states collected by F. Fedden, Esq. Journal of the Asiatic Society of Bengal 34: 273–279.

[B120] Theobald W (1876) Descriptions of some new land and freshwater shells from India and Burma. Journal of the Asiatic Society of Bengal 45: 184–189.

[B121] Tomlin JR le B (1938) New Malay land shell. Journal of Conchology 21: 73–75.

[B122] Troschel FH (1847) Ueber die Gattungen der Cyclostomiden. Zeitschrift für Malakozoologie 4: 42–45.

[B123] Tumpeesuwan S Tumpeesuwan C (2015) First record and description of a new species of the land snail genus *Pearsonia* Kobelt, 1902 (Cyclophoridae: Pterocyclinae) from Thailand, with a note on radula morphology. Raffles Bulletin of Zoology 63: 287–292.

[B124] Vermeulen JJ (1996) Notes on terrestrial molluscs of Java, Bali and Nusa Penida. Basteria 59: 149–162.

[B125] Vermeulen JJ Whitten AJ (1998) Fauna Malesiana Gude to the Land Snails of Bali. Backhuys Publisher, Leiden, The Netherlands, 164 pp.

[B126] Welter-Schultes FW (1999) Systematisches Conchylien-Cabinet von Martini und Chemnitz (1837–1920), bibliography of the volumes in Göttingen. Archives of Natural History 26: 157–203. 10.3366/anh.1999.26.2.157

[B127] Wenz W (1938–1944) Teil 1: Allgemeiner Teil und Prosobranchia. In: Schindewolf OH (Ed.) Handbuch der Paläozoologie, Band 6, Gastropoda, Verlag Gebrüder Bornträger, Berlin, 1639 pp.

[B128] Zilch A (1955) Die Typen und Typoide des Natur-Museums Senckenberg, 15: Mollusca, Cyclophoridae, Cyclophorinae-Cyclophoreae (2). Archiv für Molluskenkunde 84: 183–210.

[B129] Zilch A (1956) Die Typen und Typoide des Natur-Museums Senckenberg, 17: Mollusca, Cyclophoridae, Cyclophorinae-Cyclophoreae (3). Archiv für Molluskenkunde 85: 33–54.

